# The car tank lid bacteriome: a reservoir of bacteria with potential in bioremediation of fuel

**DOI:** 10.1038/s41522-022-00299-8

**Published:** 2022-04-28

**Authors:** Àngela Vidal-Verdú, Daniela Gómez-Martínez, Adriel Latorre-Pérez, Juli Peretó, Manuel Porcar

**Affiliations:** 1 Institute for Integrative Systems Biology I2SysBio (University of Valencia – CSIC), Calle Catedrático Agustín Escardino Benlloch 9, 46980 Paterna, Spain; 2 Darwin Bioprospecting Excellence S.L, Calle Catedrático Agustín Escardino Benlloch 9, 46980 Paterna, Spain; 3 Department of Biochemistry and Molecular Biology, University of Valencia, Calle Dr. Moliner 50, 46100 Burjassot, Spain; 4 Present Address: Department of Biological and Environmental Sciences, University of Gothenburg, Carl Skottsbergs gata 22B, 41319 Gothenburg, Sweden

**Keywords:** Microbial ecology, Applied microbiology

## Abstract

Bioprospecting of microorganisms suitable for bioremediation of fuel or oil spills is often carried out in contaminated environments such as gas stations or polluted coastal areas. Using next-generation sequencing (NGS) we analyzed the microbiota thriving below the lids of the fuel deposits of diesel and gasoline cars. The microbiome colonizing the tank lids differed from the diversity found in other hydrocarbon-polluted environments, with *Proteobacteria* being the dominant phylum and without clear differences between gasoline or diesel-fueled vehicles. We observed differential growth when samples were inoculated in cultures with gasoline or diesel as the main carbon source, as well as an increase in the relative abundance of the genus *Pseudomonas* in diesel. A collection of culturable strains was established, mostly *Pseudomonas*, *Stenotrophomonas*, *Staphylococcus*, and *Bacillus* genera. Strains belonging to *Bacillus*, *Pseudomonas, Achromobacter*, and *Isoptericola* genera showed a clear diesel degradation pattern when analyzed by GC-MS, suggesting their potential use for bioremediation and a possible new species of *Isoptericola* was further characterized as hydrocarbon degrader.

## Introduction

Fuel leaks and spills are one of the main causes of soil and groundwater contamination^1^. Given their hydrophobic nature, these pollutants are very recalcitrant compounds that constitute a serious threat to the quality of soils and water bodies^2^. From ocean spills to leaks from pipes or gas stations in highly urbanized areas, fuels can definitely seed irreversible ecological damage that cannot be overlooked^3^.

When a leak occurs, these complex hydrocarbons are released into the environment in concentrations that generally exceed the ecological resilience of the ecosystem^4,5^. Bioremediation exploits the detoxification pathways of living organisms as a tool to remove pollutants from the environment^6^. Bacteria have evolved for billions of years, and, as a result, they have developed a very diverse range of metabolic pathways that makes them capable of obtaining energy from virtually every organic compound^7^. Their ubiquity in nature, metabolic diversity, high growth rates, and their ability for horizontal gene transfer, shapes them into perfect candidates for bioremediation of pollutants, including fuels^7,8^.

It is not surprising that bacteria are known to inhabit highly oil-polluted areas. Indeed, many described bacteria of interest for bioremediation of oil spills and fuel leakages have been isolated from hydrocarbon-polluted soils, sediments, or water^9–12^. For the biodegradation of highly hydrophobic pollutants, a key feature shown by most bacterial strains is the production of extracellular compounds, known as biosurfactants, which assist in the cleavage of the parent compound, and increase the bioavailability, overall helping in the emulsification of hydrocarbons^13^.

There are numerous studies that demonstrate the biodegradation of compounds such as polycyclic aromatic hydrocarbons (PAHs) by bacteria belonging to the phylum *Proteobacteria*, isolated from water^14^ or polluted soils^15^. The majority of the formally described genera of hydrocarbon-degrading bacteria fall within this very large phylum of Gram-negative organisms^16^. Another group of pollutants with a hydrocarbon nature are the carcinogenic polychlorinated biphenyls (PCBs), which are also degradable by bacteria belonging to the *Proteobacteria* phylum^17,18^. Within this large group of contaminants susceptible to biodegradation, gasoline and diesel are not exceptions. Many bacterial species have been found to have a degrading capacity on these compounds^16,19–22^.

A great part of the bioprospecting activities performed to date to isolate bacterial fuel degraders is based on the research of the microorganisms naturally inhabiting polluted soils, water, or air^10,23–25^. Despite their overwhelming use today, there are not, to the best of our knowledge, studies on the bioremediation potential of car-associated microorganisms. Combustion cars are still the most used ones, but they have not previously been seen as a source of biotechnologically relevant bacteria. The surface of automobile tank lids is an environment that is in contact with the fuel and usually is subjected to high temperatures. In addition, it is in contact with the outside, being an ideal scenario to find and isolate bacteria with biosurfactant activity, that are already adapted to conditions where the only direct carbon source they receive is the hydrocarbon with which the car is powered, usually either gasoline or diesel. Therefore, the car tank microbiota holds potential as a bioremediation tool for environments contaminated by spills and/or prolonged exposure to diesel and/or gasoline. We present here a complete characterization of the bacteriome of a previously unexplored microbial niche, the car tank lid; and describe a laboratory selection strategy through which we have identified several bacterial strains with a remarkable ability to biodegrade diesel.

## Results

### 16S rRNA gene-based microbial community analyses

In order to study the bacterial taxonomic profile of the tank lids, we sequenced 19 samples out of the original 20 samples. Ten of them came from vehicles fed with diesel, and the other nine, fed with gasoline (sample 17G was discarded due to very low amounts of DNA in it). Rarefaction curves showed that all the samples were sequenced at a proper sequencing depth (Supplementary Fig. 1), 1,206,073 reads and 4755 amplicon sequence variants (ASVs) were obtained after quality filtering and chimera removal. The average read count per sample was 54,822 (min: 16,633; max: 121,789).

**Fig. 1:**
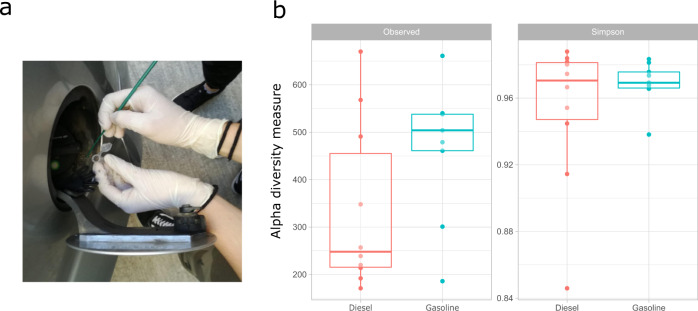
Sampling procedure and α-diversity indexes. **a** Sampling was carried out by scrapping the dust accumulating below the tank lid and around the filling point. **b** Alpha diversity metrics at the ASV level: observed ASVs -or richness- (left panel) and Simpson index (right panel) for all the samples grouped by type of fuel. ASV amplicon sequence variant. Centerline = median; bounds of box = Q1 and Q3; lower whisker = smallest observation greater than or equal to lower hinge—1.5 * IQR; higher whisker = largest observation less than or equal to upper hinge + 1.5 * IQR; Q quartile, IQR interquartile range.

Both richness and diversity (Simpson’s index) were similar in gasoline and diesel tank lids (Fig. 1b; *p* value > 0.1 two-sided Mann–Whitney *U-*test).

**Fig. 2:**
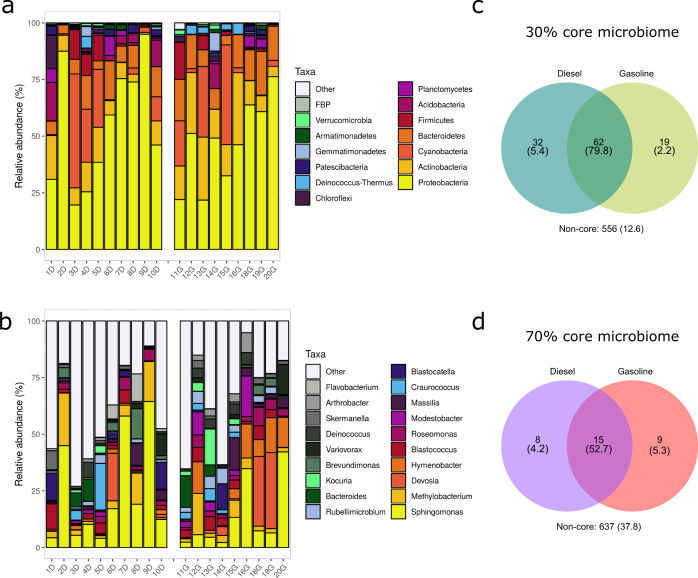
Diversity of bacterial communities from car tank lids fueled with either diesel or gasoline. Taxonomic profiles at the phylum (**a**) and genus (**b**) level resulting from the DNA extraction of the 19 samples from car tank lids. D indicates samples from diesel-fueled cars and G samples from gasoline-fueled cars. **c**, **d** Core microbiome analysis and comparison between diesel and gasoline samples. A 30% sample representation threshold, as well as 70%, is shown. Numbers indicated in parentheses correspond to %.

In general, tank lid bacteriomes proved variable and were similar in gasoline-fed and diesel-fed cars. Principal Coordinates Analysis (PCoA) confirmed that there is not a significant difference between the gasoline-fueled and diesel-fueled vehicles in terms of the microbial community that inhabits their tank lids (Supplementary Fig. 2; *p* value > 0.1, PERMANOVA test). The mean relative abundance for each phyla and genera was calculated and the most abundant ones are shown in Fig. 2a, b and Supplementary Fig. 3. In terms of phyla (Fig. 2a), the bacteriomes were dominated by *Proteobacteria* (Diesel (D): 55.2%; Gasoline (G): 47.2%), followed by *Actinobacteria* (D: 9.1%; G: 16.7%), *Cyanobacteria* (D: 11.7%; G: 11.4%), *Bacteroidetes* (D: 8.3%; G: 13%), *Firmicutes* (D: 3.8%; G: 3.1%), and *Acidobacteria* (D: 4%; G: 1.4%). At the genus level (Fig. 2b), *Sphingomonas* (D: 24%; G: 13.2%) turned out to be rather frequent in all the samples. This genus together with *Methylobacterium* (D: 7.1%; G: 4.7%)*, Devosia* (D: 2.4%; G: 7.7%)*, Hymenobacter* (D: 1%; G: 8%)*, Blastococcus* (D: 3%; G: 3.8%)*, Roseomonas* (D: 2.2%; G: 3.4%)*, Modestobacter* (D: 0.7%; G: 4.3%)*, Massilia* (D: 1.9%; G: 3.1%)*, Craurococcus* (D: 2.7%; G: 1.8%), *and Blastocatella* (D: 2.9%; G: 1.4%) constitute the ten most abundant genera in car tank links. Furthermore, the class distribution among the *Proteobacteria* phylum was also obtained (Supplementary Fig. 4). *Alphaproteobacteria* was by far the most abundant class in both diesel and gasoline-fueled cars (D: 44.2%; G: 39.6%). *Gammaproteobacteria* (D: 10.0%; G: 7.0%) and *Deltaproteobacteria* (D: 1.0%; G: 0.4%) were also present in car tank lid bacteriomes.

**Fig. 3:**
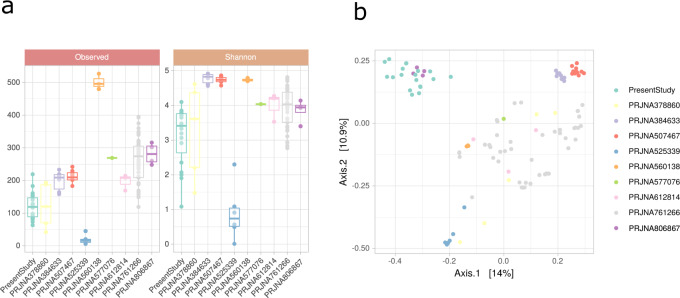
Taxonomic profile of the car tank lids compared to hydrocarbon-polluted soils and urban solar panels. Bioproject numbers “PRJNA525339 and PRNJA378860” correspond to microbial communities developed in cultures and soil, respectively, after the enrichment with a sole type of hydrocarbon. Bioproject numbers “PRJNA384633, PRJNA761266, PRJNA577076, PRJNA612814, PRJNA560138, and PRNJA507467” belong to studies on the natural microbial community in hydrocarbon-polluted soils. “PRJNA806867” corresponds to samples where the microbiota on solar panels was studied. More detailed information about each Bioproject and BioSamples can be found in Supplementary Dataset 1. **a** Alpha diversity metrics at the ASV level: observed ASVs -or richness- (left panel) and Shannon index (right panel) for all the selected datasets found in the literature and the dataset of the present study. ASV amplicon sequence variant. **b** Principal component analysis based on the beta diversity at a genus level. Each point corresponds to one BioSample (Description of each sample and accession numbers in Supplementary Dataset 1).

A core microbiome was calculated in order to compare the bacterial communities from both, gasoline-fueled cars and diesel-fueled vehicles. The car tank lid microbiome at 30, 50, and 70% sample representation (taxa above 0.1% in abundance present in the 30, 50, or 70% of the samples, respectively) is shown in Fig. 2c, d (30 and 70%) and Supplementary Fig. 5 (50%). At 30% sample representation, 79.8% of the taxa were common among gasoline and diesel car tank lids and contained 62 different genera. As expected, the taxa in common decreased to 52.7% when the sample representation threshold was risen to 70% and comprised only 15 genera. Also, regarding genera that were only present significantly in the bacterial community of one fuel type, at the 70% sample representation cut-off, eight and nine differential genera belonged only to the diesel or gasoline core microbiome, respectively. In detail, the genera *Pedobacter, Novosphingobium, Flavobacterium* and other genera from *Armantimonadales, Rhizobiales, Saccharimonadales*, and *Micrococcales* orders constituted the eight unique genera in the diesel core-microbiome. In the same way, *Cnuella, Adhaeribacter, Deinococcus, Geodermatophilus, Noviherbaspirillum, Cellulomonas, Friedmanniella, Arthrobacter* genera, and some uncultured genera from the *Acetobacterales* order were present significantly in the 70% of the gasoline samples and not in diesel samples. Nevertheless, differential abundance analyses only showed three genera significantly represented in diesel samples (*Flavobacterium*, *Muricoccus*, and uncultured bacteria belonging to *Limnochordaceae* family) probably due to the high variability observed in the microbiome composition.

### Taxonomic profile of the car tank lids compared to other hydrocarbon-polluted environments

Alpha diversity in the car tank lid bacteriome (Fig. 3a) proved to be lower compared to other hydrocarbon-polluted soil environments (Supplementary Dataset 1). As could be expected, principal coordinates analysis (PCoA), revealed the differences in the microbial communities found in soil polluted environments and in the surface of urban artificial environments (i.e., car tank lids and solar panels) (Fig. 3b). Nevertheless, a core microbiome of all the samples exposed to hydrocarbon pollution showed that *Pseudomonas* and *Sphingomonas* were present in at least 90% of the samples with an abundance higher than 0.001%.

### Tank lid biomass culture with hydrocarbon enriched cultures

**Fig. 4:**
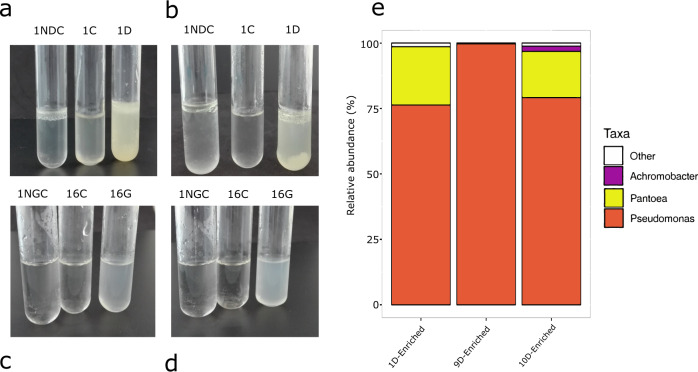
Decreased diversity of car tank lid samples in enriched cultures with gasoline or diesel as the sole carbon source. Hydrocarbon-containing enriched cultures resulting from the inoculum from vehicle 1 (diesel) (**a**, **b**) and vehicle 16, fueled with gasoline (**c**, **d**) after a week of incubation in a chamber and shaking at 30 °C, in the dark (**a**, **c**); and after four consecutive weekly passes under the same incubation conditions (**b**, **d**). 1NDC negative diesel control replica 1; 1C control minimal medium without fuel, inoculated with sample 1D; 1D diesel medium inoculated with sample 1D. 1NGC negative gasoline control replica 1; 16 C control minimal medium without fuel, inoculated with sample 16 G; 16 G gasoline medium inoculated with sample 16 G. **e** Genus-level taxonomy of three enriched diesel samples.

After 4 weeks of incubation and increasing the gasoline or diesel concentration each week (by means of a new inoculation in fresh media with the corresponding fuel), microbial consortia capable of surviving under a high concentration of diesel or gasoline were selected (Fig. 4a–d).

In general, the cultures with gasoline as the sole carbon source had a lighter, whitish, and more homogeneous appearance compared to media with diesel, which turned out to be emulsified in little drops. Microbial growth could be easily visualized through the turbidity of the liquid cultures. The group including samples 13G, 15G, 16G, 17G, 18G, and 20G showed very little growth during the first week, but a remarkable growth after 4 weeks. Some cultures exhibited a steady growth throughout the duration of the experiment, as is the case of cultures 12G and 14G. The 19G sample showed no obvious growth, and 11G showed the highest growth after the first week. In cultures with diesel, samples 1D, 9D, and 10D yielded a higher growth. Nevertheless, 3D, 4D, 7D, and 8D showed moderate growth at all times. Finally, no growth was observed in some samples (2D, 5D, and 6D).

### Microbial communities from enriched cultures

In order to compare the initial microbial communities from the car tank lids, with the enriched cultures after 4 weeks under selection pressure, the DNA was extracted from the samples that turned out to have a clear growth during the enrichment procedure. It was not possible to isolate DNA from gasoline-containing samples, hence, we decided to continue with the samples that showed a higher growth from diesel enriched cultures (1D, 9D, and 10D). The microbiome analysis of the samples supplemented with diesel displayed saturated rarefaction curves (Supplementary Fig. 1). As expected, enriched samples with diesel as the sole carbon source resulted in a decreased degree of richness and diversity in comparison to the original tank lid samples from the diesel-powered vehicles (Supplementary Fig. 6).

PCoA analyses showed a clear separation between the enriched samples and the initial ones (Supplementary Fig. 6; *p* value < 0.005, PERMANOVA test).

Regarding taxonomic composition (Fig. 4e), *Pseudomonas* spp. predominated in the three enriched samples, with more than the 75% abundance of the total microbiome in all of them. Also, *Pantoea* (17% in two samples) and *Achromobacter* with the 2% abundance in one sample, were present in the enriched microbial populations.

### Bacterial collection characterization

A collection of bacterial strains from the enriched cultures was set up, by inoculating culture aliquots on LB and R2A agar plates. After two weeks, colonies with different phenotypes were observed in all the plates from diesel enriched cultures. On the other hand, microbial growth was only detected in three gasoline-enriched samples (11G, 15G, and 19G).

A total of 48 different colonies were isolated and named with a code, according to the number of the vehicle sampled and the type of fuel (G: gasoline car, D: diesel vehicle) followed by a number designating the colony picked in each plate. Finally, 47 of them could be identified through 16S rRNA gene sequencing (Supplementary Table 1). A higher diversity of bacteria was found in the media containing diesel. In agreement with the results obtained through NGS, in diesel cultures, *Pseudomonas* (24%) was the most frequent genus, followed by *Stenotrophomonas* (16%), *Achromobacter* (11%), *Acidovorax* (8%), *Isoptericola* (8%), *Cellulosimicrobium* (8%), *Paenibacillus* (5%), *Bacillus* (5%), *Pantoea* (5%), *Cellulomonas* (3%), *Skermanella* (3%), *Agrobacterium* (3%), and *Sphingobium* (3%) (Supplementary Fig. 7).

On the other hand, the nine strains isolated from cultures supplemented with gasoline belonged to only five genera, where *Staphylococcus* and *Bacillus* were the most frequent genera (33%), followed by *Microbacterium* (11%), *Isoptericola*, and *Kocuria* (11%) (Supplementary Fig. 7).

### Selection of surfactant-producing strains

**Fig. 5:**
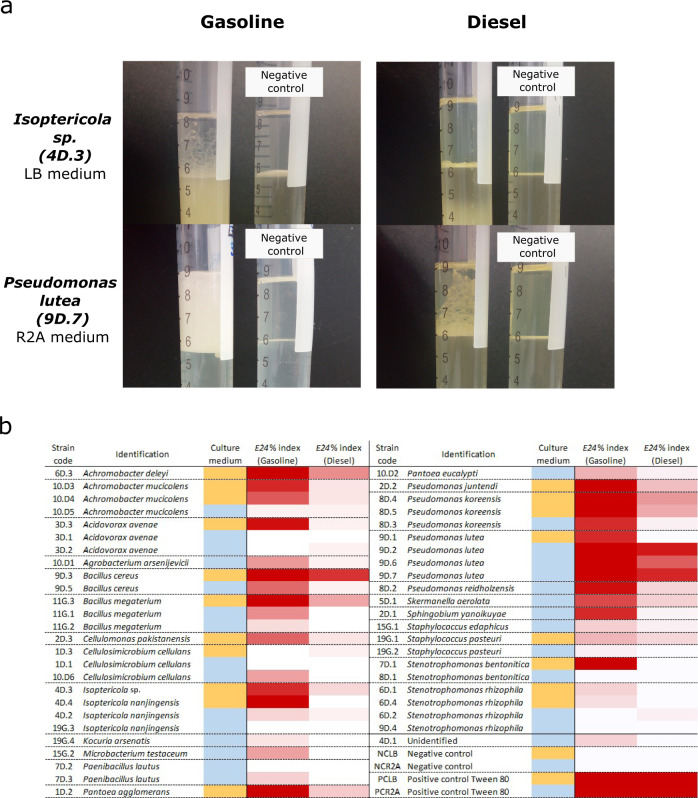
Emulsification activity by strains in the collection. To carry out this assay, each strain was propagated in LB or R2A liquid medium depending on the medium from which they were isolated at first, then they were mixed with gasoline and diesel. **a** Assay tubes where the emulsifying effect of strains *Isoptericola* sp. 4D.3 and *Pseudomonas lutea* are shown in contrast to control tubes with fresh medium and non-inoculated bacteria. **b** Heat map of the emulsification index (*E*_24_%). In a red gradient, the ability to emulsify the fuel is shown, from dark red (highest *E*_24_%*)* to white (no emulsifying effect). The medium where each strain was grown is displayed in orange (LB) and blue (R2A). Negative controls in both medium (NC.LB; NC.R2A), as well as positive controls with Tween 80 as a surfactant (PC.LB; PC.R2A), showed no emulsifying effect and complete emulsification of the fuel layer, respectively.

The production of biosurfactants was assessed by the emulsification index (*E*_24_%), which measures the ability of the strains to emulsify the hydrophobic phase and consequently the possibility of the bacterial cell to be in contact directly with the fuel to degrade it. The calculated *E*_24_% for each strain (that had previously grown in the pertinent isolation medium, emulsifying not only diesel but also gasoline) is shown in Fig. 5. In general terms, a clear difference due to the type of fuel was observed, as well as the differential behavior regarding the medium in which the preculture was grown. While just a few isolates could emulsify diesel successfully, a huge proportion of the strains in the collection were able to emulsify gasoline. Furthermore, as some of the isolates finally belonged to the same species, a pattern of emulsifying power linked to the nutrient concentration of the growth medium, rich (LB) or poor (R2A), was also observed. Usually, the same species that were grown in LB instead of R2A, yielded a higher *E*_24_% as they could reach a higher OD_600,_ except *Pseudomonas lutea* that showed the opposite effect. Five isolates (4D.3, 6D.3, 8D.2, 9D.7, and 11G.3), that showed a high *E*_24_% were selected to perform further mass spectrometry analysis in order to verify and quantify their ability to degrade hydrocarbons. These selected strains belonged to the genus *Pseudomonas* (2), *Bacillus*, *Isoptericola*, and *Achromobacter*.

### Diesel biodegradation by bacterial strains

**Fig. 6:**
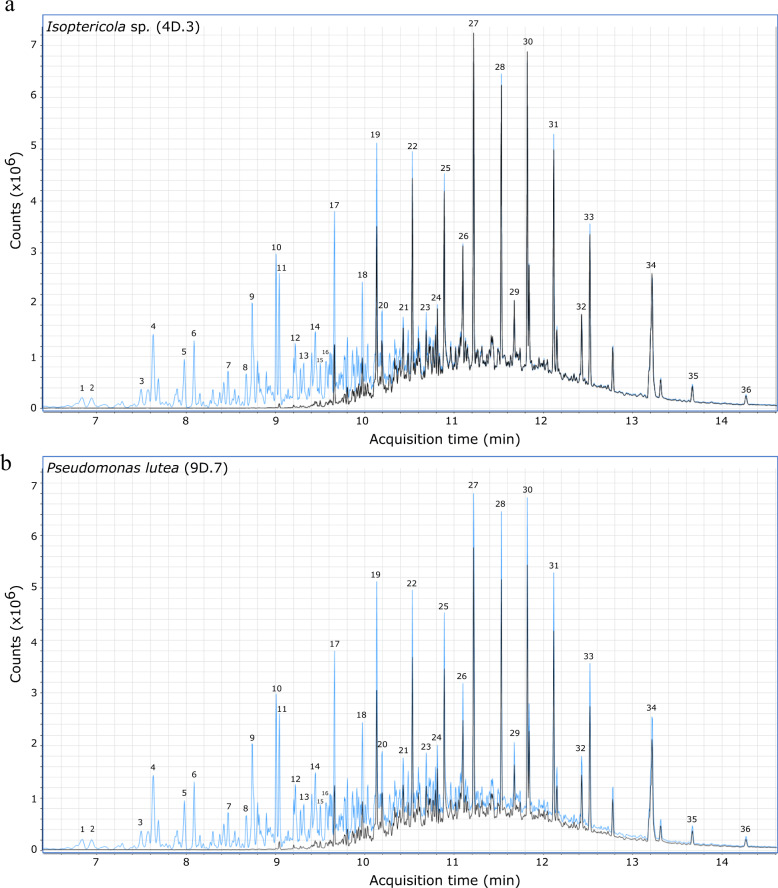
Gas chromatograms of diesel extracted from cultures inoculated with putative hydrocarbon-degrading bacteria. The non-degraded diesel profile (in blue) compared to the degraded diesel profile (black) when it was incubated for 7 days at 30 °C in BH medium with a selected hydrocarbon-degrading strain from the collection: **a** *Isoptericola* sp. (4D.3); **b** *Pseudomonas lutea* (9D.7). Each peak refers to a compound which was identified by comparing the mass spectra with commercial libraries and they are identified in Supplementary Table 2.

**Fig. 7:**
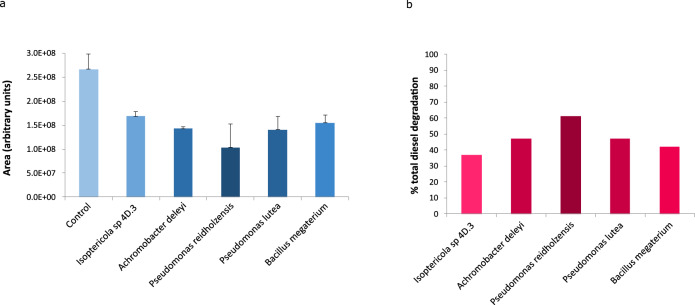
Total diesel degradation by isolates. **a** Calculated area under the chromatogram of each sample, the average and the standard deviation of the three replicates is shown. **b** Percentage of degradation by each isolate taken as 100% of the total area of control samples (not subjected to microbial degradation). Higher color intensity relates to higher diesel-degrading activity.

In order to identify possible diesel-degrading activities from the bacterial collection, the strains 4D.3 (*Isoptericola sp*.), 6D.3 (*Achromobacter deleyi*), 8D.2 (*Pseudomonas reidholzensis*), 9D.7 (*Pseudomonas lutea*), and 11G.3 (*Bacillus megaterium*), with a high *E*_24_%, were subjected to the growth on minimal medium supplemented with diesel as sole carbon source and finally the remaining diesel after 1-week incubation was analyzed by GC-MS. The experiment was conducted in triplicate and all the replicates within the same sample showed a very similar pattern (Supplementary Fig. 8). All the selected strains caused the first peaks in the diesel chromatography profile to disappear (peaks 1 to 18), the ones that correspond to small aromatic hydrocarbons and shorter hydrocarbon molecules (C_6_-C_13_) (see Supplementary Table 2 for the identification of compounds), meanwhile, in the control sample, all of these first peaks were still present after 1-week incubation. In the same way, the largest alkanes and alkenes (C_15_-C_20_), corresponding to peaks with higher retention time (peaks 19 to 36), remained in both sample types (controls and inoculated cultures), showing a slight decrease in some isolates (Fig. 6). In particular, comparing areas in the chromatograms (Fig. 7), *Pseudomonas reidholzensis* showed the highest global degradation of diesel (61%), followed by *Achromobacter deleyi* and *Pseudomonas lutea* (47%), all of them displaying to some extent a decrease in peaks with higher retention time. Meanwhile, *Isoptericola* sp. 4D.3 and *Bacillus megaterium* were limited to the degradation of smaller compounds and showed a total diesel degradation of 37 and 42% respectively.

*Isoptericola* sp. 4D.3 genome was sequenced in order to elucidate the metabolic pathways involved in hydrocarbon degradation. Genomic distances between the sequenced genome and all the genomes available for the genus *Isoptericola* were calculated to assess the taxonomic affiliation of the strain. Interestingly, both the Average Nucleotide Identity (ANI) and the digital DNA-DNA hybridization (dDDH) values were below 95 and 70%, respectively, which are the thresholds for defining new species^26,27^. Therefore. Strain 4D.3 probably constitutes a new species within the genera *Isoptericola*, having as closest strain *Isoptericola nanjingensis*, according to the 16S rRNA gene. The functional annotation of the genome by BlastKoala revealed the presence of several enzymes involved in xenobiotic degradation pathways and a complete degradation pathway of aromatic compounds to succinyl-CoA and acetyl-CoA was annotated (Supplementary Fig. 9 and Supplementary Dataset 2). In the same way, some enzymes are known to be the starting point of aromatic and alkane hydrocarbon degradation pathways, such as mono- and dioxygenases, were found in *Isoptericola sp*. 4D.3 (Supplementary Dataset 3). Remarkably, using the CANT-HYD database^28^, enzymes from both aerobic and anaerobic hydrocarbon degradation pathways were found in this strain.

## Discussion

In this work, we have characterized the bacterial communities that thrive in car tank lids from a dual perspective. On the one hand, a culture-independent microbiome analysis comparing the original bacteriome from diesel and gasoline-fueled cars as well as with other hydrocarbon-polluted environments. On the other hand, a culturomics approach in order to detect culturable fuel-degrading bacteria with potential for fuel bioremediation.

Our results show that the diversity of the inhabiting bacteriomes in car tank lids is not driven by the type of fuel with which the car is fed. A slightly lower diversity is observed in the tank lid bacteriomes when comparing them to bacteriomes from polluted soils and solar panels (see Supplementary Dataset 1 for a list of the studies included in the meta-analysis). These findings suggest that diversity might be driven by an ecological niche question more than the main type of carbon source question. Interestingly, car tank lids and solar panels tend to group together in terms of bacterial composition and seem to differ from the soil hydrocarbon-polluted bacteriomes. Therefore, it could be hypothesized that those microbiomes thriving on artificial, urban surfaces appear to be already exposed to previous selection pressure, such as high temperatures, dryness, and lack of nutrients than might be more available in the soil environment. Furthermore, these microbial communities originated from air-borne environmental inoculums that probably contain a mix of microorganisms and other particles, such as small combustion products, which can potentially increase the chances of finding hydrocarbon-degrader organisms in these environments. Moreover, the selection pressure due to the low nutrient environment rich in hydrocarbons may explain the decrease in diversity in the car tank lids compared to the other samples included in the analysis.

In any case, our results are in line with previous findings in the literature where *Proteobacteria* is usually the predominant phylum in fuel-contaminated samples^12,29^. In the same way, *Bacteroidetes, Actinobacteria, Firmicutes*, and even *Cyanobacteria* have been described as abundant phyla in diesel car tanks^29^ as well as oil-spilled beaches^12,30^. The same pattern can be observed at the genus level, being remarkable the ubiquitous presence of *Sphingomonas* in all the samples. This genus has previously been described in very diverse fuel-polluted environments, from samples in the Antarctic^31,32^, too polluted soils in gas stations^33^. It has been described deeply as a hydrocarbon-degrading bacteria that can degrade a wide variety of petroleum derivatives^31,34^, especially diesel^35,36^. In particular, the genera *Sphingomonas* has been widely described to degrade efficiently aromatic compounds, due to the presence of intertwined catabolic pathways for the degradation of both monocyclic and polycyclic aromatics^36–39^. For instance, the *Sphingomonas* strain Ant 17 was proved to efficiently degrade the aromatic fraction of diesel in a similar way as the five selected diesel-degrading strains did in this study^31^. *Methylobacterium* is also a genus present in almost all the samples and it has been described, together with *Sphingomonas*, to be the first microbial colonizers of stored fuels^40^. Other less abundant genera such as *Variovorax* and *Massilia* have already been described as capable of surviving in hydrocarbon-containing samples, such as diesel, maintaining its cell structure and functional integrity^41,42^. Therefore, our findings are in agreement with previous descriptions of bacterial communities from diverse fuel-containing environments, showing the establishment in the previously unexplored niche of the fuel tank lid of a characteristic microbiota driven by oil-derived components.

A laboratory selection procedure of the initial microbial community pool was carried out, which allowed setting a collection of putative diesel- or gasoline-degrading bacteria. It has to be stressed that most of the previous studies enriched their cultures with fuel-degrading bacteria using around 5% of hydrocarbon of the total volume^23,43,44^, whereas we prepared the cultures with 10% of gasoline or diesel from the total volume. The aim of establishing an enriched culture was, on one hand, the identification of putative culturable oil-degrading bacteria. On the other hand, we aimed at identifying taxonomic changes in the enriched cultures compared to the initial samples through NGS. As expected, there was a reduction of diversity in the microbial community after several weeks in cultures with oil (diesel or gasoline) as the sole carbon source. *Pseudomonas* was by far the most abundant genus in diesel cultures as well as in the selected strains in the collection isolated from diesel car tank lids. This substantiates previous findings in the literature where *Pseudomonas* has been characterized as the main alkane and hydrocarbon polycyclic aromatic degrader genus^45^. Thanks to the metabolic diversity of *Pseudomonas*, this species is used for the bioremediation of soils contaminated with organic compounds, such as hydrocarbons^46^, naphthalene^47^, or herbicides^48^, among others. However, it is remarkable that the genus *Pseudomonas* in the initial microbial community in the car tank lid was undetectable, suggesting that, in the sampled environment, it is present at a very low abundance. In two of the cultures supplemented with diesel, the Gram-negative genus *Pantoea* was also abundant. Interestingly, alkane-degrading genes have been described in both *Pantoea* and *Pseudomonas* genera (*alkB* gene and alkane hydroxylase (CYP153) gene respectively), and they have been used in bioremediation of diesel contaminated soil^49^.

Unfortunately, no DNA from enriched cultures supplemented with gasoline could be obtained. Nevertheless, several strains in the collection of culturable bacteria did originate from car tank lids of gasoline cars, showing a differential set of Gram-positive bacterial strains in comparison to strains that were isolated from diesel cars, which were mostly composed of Gram-negative species. In contrast with the *Pseudomonas*-rich diesel enriched microbiomes, *Staphylococcus* and *Bacillus* constituted almost 80% of all the strains isolated from gasoline cars. This fits with previous findings on both genera being able to survive and grow in gasoline-contaminated soils^50^. Furthermore, *Bacillus megaterium*^51^, *Kocuria* sp.^52^, *Staphylococcus saprophyticus*^53^ have been described as degraders of methyl-*tert*-butyl ether (MTBE) which is a gasoline additive highly used since the 20th century and that has turned out to pollute the soils^52^. Interestingly, all the strains isolated from gasoline-car tank lids were Gram-positive and have been described as MTBE degrading bacteria.

Hydrocarbons are very recalcitrant in the environment largely because of their low bioavailability, since they are very hydrophobic compounds. Biosurfactants are amphipathic molecules, with hydrophobic and hydrophilic moieties, capable of reducing the superficial tension, and thus significantly enhancing the solubility, motility, bioavailability, and, hence, biodegradation of hydrophobic compounds^54^. Biosurfactant production is considered a key factor of bacterial development in the presence of fuels as it gives the capability to emulsify hydrocarbons and therefore be able to degrade them^55^. A wide range of genera are capable to synthetize these molecules^56,57^, in particular, *Pseudomonas* and *Bacillus* have been widely described as biosurfactant producers^58,59^. For example, rhamnolipids are glycolipid-type biosurfactants produced mainly by *Pseudomonas* species that have been used for a wide range of applications in the industry and bioremediation of oil pollutants because of their high emulsification capability^56,60,61^. In the same way, *Bacillus* species which have shown high biosurfactant production in this study, have been described as biosurfactants producers that effectively enhance diesel biodegradation by themselves as well as other hydrocarbon-degrading strains^33^. Furthermore, the biosurfactant production of *Pantoea*, a genus detected in the three enriched samples, was reported by Walterson & Stavrinides (2015)^62^ and some species belonging to *Isoptericola* genera, such as *Isoptericola chiayiensis* have also been described as rhamnolipid producers^63^. Moreover, it is interesting to highlight that richer media increased the biosurfactant production by the strains in our collection.

Therefore, there is a straightforward link between biosurfactant production and fuel biodegradation, which has been confirmed by GC-MS in this study. The best five emulsifier strains we isolated and characterized showed robust growth in the presence of diesel in a minimal medium and displayed a similar diesel degradation pattern, being capable of completely degrading small alkanes and aromatic hydrocarbons C_6_-C_13_ and partially degrading larger alkanes and alkenes C_15_-C_20_ after 1 week of incubation with diesel as the sole carbon source. Diesel is a complex mixture of linear and cyclic hydrocarbons which have been described to range from C_8_ to C_26_, being C_10_ to C_20_ -hydrocarbon chains the most abundant ones^64^. According to these results and the existing bibliography, it could be hypothesized that longer incubation periods could have led to an increase in larger hydrocarbon degradation^65–67^. Hydrocarbon degradation, in particular diesel compounds, by *Pseudomonas* spp*., Bacillus* spp., and *Achromobacter* spp. has been widely studied^33,41,64,68^. However, to the best of our knowledge, *Isoptericola* has been sparsely studied as hydrocarbon-degrading bacteria^63,69^. Hence, our study provides considerable insight into the potential of the new *Isoptericola* sp. 4D.3 strain for hydrocarbon bioremediation. The complete genome sequencing of this strain revealed a metabolic repertoire within aromatic and aliphatic hydrocarbon degradation in both aerobic and anaerobic pathways. These findings are in good agreement with the previous literature as the type strain of this genus, *Isoptericola variabilis*, has been described as a facultative anaerobic microorganism^70^. Nevertheless, further analyses to describe this potentially new species as well as to completely outline these degradation pathways are needed.

Taken together, our results show that car tank lids are specific micro niches for microbial life that differ from oil-polluted soils, which have been traditionally used to isolate hydrocarbon-degrading microorganisms. In this study, we have proved that they contain bacterial isolates, even new species, which hold great potential for fuel biodegradation of diesel or gasoline-contaminated environments.

## Methods

### Sampling

The samples were collected at the parking areas of the Institute for Integrative Systems Biology (I^2^SysBio. Paterna, Spain) and the Polytechnic University of Valencia (Valencia, Spain), between the second and third of July 2018. The procedure followed to collect the dust accumulated in the deposits consisted of the election of 20 random vehicles, whose owners agreed to participate in the study. Ten vehicles were fueled with diesel (1D–10D) and another ten were fueled with gasoline (11G–20G). All vehicles were older than 1 year.

By using a sterile handle, the deposits were “scratched” to obtain a black powder or putty consisting of dust totally or partially soaked with fuel residues (Fig. 1a). Once obtained, samples were deposited in 1.5 mL sterile tubes and transported to the Institute for Integrative Systems Biology (I2SysBio) for further processing steps.

### 16S rRNA gene sequencing and analysis

DNA extraction of the tank lid biomass was performed with the PowerSoil^®^ DNA Isolation Kit (MoBio Laboratories, Inc., CA, USA, 12888-100). From each vehicle, 0.2 g of biomass were used. For the validation of the extraction a PCR was performed, with the primers, 18 F (5′-CACCAGGTTGATTCTGCC-3′) and 1537 R (5′-TTATGATCCTGCTAATGGTTC-3′), amplifying a region of the 16S rRNA gene, followed by electrophoresis in a 1.4% (w/v) agarose gel.

The V3–V4 region (459 bp) of the 16S rRNA gene was amplified in all the samples following the Illumina MiSeq’s protocol and using the recommended primers: “16S Amplicon PCR Forward Primer” (5′-TCGTCGGCAGCGTCAGATGTGTATAAGAGACAGCCTACGGGNGGCWGCAG-3′) and “16S Amplicon PCR Reverse Primer” (5′-GTCTCGTGGGCTCGGAGATGTGTATAAGAGACAGGACTACHVGGGTATCTAATCC-3’). PCR amplification consisted of the following steps: (1) initial denaturation at 95 °C (3 min), (2) 25 cycles of annealing (95 °C 30 s, 55 °C 30 s, 72 °C 30 s), and extension at 72 °C (5 min), using a KAPA HiFi HotStart ReadyMix (KK2602). Then, adapters and barcodes (Nextera XT index kit v2, FC-131-2001) were added to the resulting amplicons. All the individual amplicon libraries were normalized and pooled together. In order to improve base calling during sequencing, the pool containing the indexed amplicons was loaded onto the MiSeq reagent cartridge v3 (MS-102-3003) spiked with 10% PhiX. Finally, sequencing was conducted using a paired-end, 2x300pb cycle run on an Illumina MiSeq sequencing system.

Raw reads were processed with QIIME2 (v. 2019.4)^71^. Briefly, the pipeline consisted of (1) the quality assessment via the demux plugin, (2) the error correction and the sequence variant clustering through DADA2, and (3) the assignment of the reads against the SILVA database (v. 132)^72^ using the classify-Sklearn module from the feature-classifier plugin. Principal coordinates analyses (PCoA) were carried out with phyloseq R package (v. 1.22.3)^73^ using Bray–Curtis dissimilarities, and PERMANOVA tests were calculated with vegan using the adonis function (v. 2.5-3). Rarefaction curves were also constructed in R via the iNEXT package (v. 2.0.17)^74^. The core microbiome analyses were performed with the amp_venn function from the ampvis2 package (v. 2.6.5) (10.1101/299537). Taxa with a relative abundance lower than 0.1% were excluded from the analysis. Referring to previous studies in the bibliography, a taxon was considered to be ‘core’ when it was present in at least 30, 50, or 70% of the samples of each group (gasoline and diesel)^75,76^. Finally, DESeq2 (v. 1.26.0)^77^ was used for differential abundance analyses and p-values adjusted with Benjamini–Hochberg method.

### Taxonomic profile of the car tank lids compared to other hydrocarbon-polluted environments

Datasets were obtained based on a literature search in Scopus (www.scopus.com), by using the search line: ALL (“16S” AND “Illumina”) AND (TITLE-ABS-KEY (hydrocarbon OR diesel OR gasoline OR oil AND contaminated OR polluted)), as well as a database search on the NCBI SRA by search with the following syntax: Hydrocarbon [All Fields] AND Polluted [All Fields] AND X metagenome [Organism], where X was “sediment” and “soil”. To confirm the completeness of this search, an additional Google Scholar search was carried out using the search terms “hydrocarbon”, “polluted”, and “Illumina.” The first 500 hits were reviewed for any studies that were not captured in the main searches. The aim of this search was to capture available datasets that describe hydrocarbon-polluted soils and other environmental surfaces. Only those studies that used the V3–V4 region of the 16S rRNA gene for microbiome sequencing were included in the selection (Supplementary Dataset 1). Both polluted and non-polluted soil samples were considered in the analysis. Four samples collected from solar panels located near the I2SysBio building (Paterna, Spain) were also included in the meta-analysis^78^.

Again, bioinformatic analyses were carried out with QIIME2^71^. All the samples included in a particular study or experiment were processed independently using the DADA2 plugin, as recommended by the authors of QIIME2 (https://docs.qiime2.org/2021.11/tutorials/fmt/). Taxonomy was assigned using the SILVA database (v. 138)^72^. For alpha diversity, a rarefaction to 10,000 reads per sample was performed. Samples below this threshold were removed from the analysis. Beta diversity analysis was performed as described in the previous section. Finally, control samples were removed from the analysis and a core microbiome was calculated considering all the samples exposed to hydrocarbon pollution (Supplementary Dataset 1). In this case, a taxon was considered to be ‘core’ when it was detected in at least 90% of the samples with an abundance higher than 0.001%.

### Liquid culturing with gasoline and diesel

In order to select microorganisms able to use as the only carbon source either diesel or gasoline, a culture with this selection pressure was established. Specifically, the carbon sources used in the cultures were Diesel (Diesel A) and gasoline (Shell-V Power Unleaded Petrol 95) purchased at a SHELL gas station (Polig. Ind. Obradors, Calle Fusters 26; Parc. G 9 -10, 46110 Godella, Valencia). For the use in the media, each fuel was filtered with Sterile Syringe Filter 0.2 µm cellulose acetate filters under sterile conditions and mixed with 3 mL of Minimal Medium (Composition in g/L: 2 NaNO_3_, 1 K_2_HPO_4_, 0.5 MgSO_4_·7H_2_O, 0.5 KCl, 0.5 Sucrose) up to a final concentration of 10% (v/v).

The Minimal Medium contained 0.5 g/L of sucrose in order to provide minimum energy for initial microbial growth. For each sample, control was established to which no gasoline or diesel was added, although an inoculum (aliquot of biomass from the tank lid) was included. In addition, two gasoline and two diesel controls with MM and no bacterial inoculation were included.

The cultures were brought to agitation (120 rpm) in a chamber at 30 °C. After 1 week, they were collected and stabilized on a rack for 30 min. Then, 25 μL of each culture were transferred to fresh media. This process was repeated weekly for four weeks. At the end, the final cultures were centrifuged at 9500×g for 15 min and the pellet obtained was subjected to DNA extraction by using the PowerSoil^®^ DNA Isolation Kit (MoBio Laboratories, Inc., CA, USA, 12888-100). Finally, the 16S gene sequencing and analysis was performed following the same procedure as previously described in the “16S rRNA gene sequencing and analysis” section.

### Strain collection and characterization

From every enriched culture tube, a dilution of 1:4 (v/v) was made. Specifically, 1 mL of culture was mixed with 4 mL of Phosphate Buffer Saline (PBS, composition in g/L: 8.0 NaCl, 0.2 KCl, 1.44 Na_2_HPO_4_, 0.24 KH_2_PO_4_; adjusted to pH 7.4). From here, also dilutions 10^−1^ and 10^−2^ were made, in order to inoculate them in agar plates. About 100 mL of these dilutions and direct aliquots from the enriched cultures were spread on Lysogenic Broth (LB, composition in g/L: 10.0 tryptone, 10.0 NaCl, 5.0 yeast extract, 15.0 agar) and Reasoner’s 2 Agar (R2A, composition in g/L: 0.5 peptone, 0.5 casaminoacids, 0.5 yeast extract, 0.5 dextrose, 0.5 soluble starch, 0.3 K_2_HPO_4_, 0.05 MgSO_4_, 0.3 sodium pyruvate, 15.0 agar) plates. All cultures were incubated in a chamber at 30 °C in darkness, under aerobic conditions for 2 weeks. The selected colonies corresponding to different phenotypes (size, shape, and color) on both LB and R2A media were re-streaked on LB or R2A fresh plates, in order to obtain pure cultures on a solid medium, which were cryopreserved in 20% glycerol (v/v) until required.

The 16S rRNA from the isolated pure cultures was sequenced. Briefly, the pure colonies were picked with a sterile handle and a loopful of the collected biomass was deposited in PCR tubes with 100 µL of sterile distilled water. After homogenization, they were boiled at 100 °C, for ~6 min and 1 µL was used as templates in the PCRs. The isolates were identified by PCR amplification of the gene encoding the 16S rRNA, using primers 8 F (5′-AGAGTTGATCCTGGCTCAG-3′) and 1492 R (5′-GGTTACCTTGTTACGACTT-3′) as described above. Some samples failed to amplify with this primer pair. In such cases, a second PCR was carried out using primers 1055 F (5′-ATGGCTGTCGTCAGCT-3′) and 341 R (5′-CTGCTGCCTCCCGTAGG-3′).

The amplification was checked by electrophoresis in a 1.4% (w/v) agarose gel. Then, the positive amplifications were precipitated overnight, with 1:1 isopropanol (v/v) and 1:10 potassium acetate (v/v), pH 5, 3 M. The tubes were then centrifuged at 4 °C for 10 min at 13,500 × *g*. The supernatant was discarded and the pellet was washed with 70% ethanol. Finally, the amplified samples were resuspended in 20 µL of Milli-Q water. BigDye^®^ Terminator v3.1 Cycle Sequencing Kit (Applied Biosystems, Carlsbad, CA, USA) was used for labeling the amplicons. Sanger sequencing was carried out by the Sequencing Service of the University of Valencia (SCSIE). Low-quality sequence ends were manually trimmed with Trev (Staden Package, 2002). Final sequences were aligned against the EzBioCloud 16S Database^79^: https://www.ezbiocloud.net/.

### Screening for bioemulsifier production

The emulsifying potential of the strains was tested following the emulsification index (*E*_24_%) described by Cooper and Goldenberg (1987)^80^ with some modifications. Isolates in the collection were grown on liquid culture in the corresponding isolation medium (LB or R2A) at 30 °C for 48 h and 180 rpm (Fig. 5b and Supplementary Table 1). Then, 3 mL of the liquid cultures were added to 3 mL of sterile diesel, mixed at the highest speed with a vortex for 2 min, and allowed to stand for 24 h. The same protocol was followed with gasoline instead of diesel. In addition, negative controls of 3 mL of sterile LB and R2A medium mixed with the proportion of fuels as well as positive controls using 3 mL of a Tween 80 2:1 (v/v) solution instead of bacterial culture were added to the assay and continued with the same procedure. Afterward, the height of the emulsified layer (mm) and the total height of the liquid column (mm) were measured. The *E*_24_% *index* of each strain in both gasoline and diesel fuels was determined by the following equation:

$$E24\% = \frac{{{\mathrm{Height}}\,{\mathrm{of}}\,{\mathrm{emulsified}}\,{\mathrm{layer}}\,\left( {{\mathrm{mm}}} \right)}}{{{\mathrm{Total}}\,{\mathrm{height}}\,{\mathrm{of}}\,{\mathrm{liquid}}\,{\mathrm{column}}\,\left( {{\mathrm{mm}}} \right)}} \times 100$$

### Gas chromatography and mass spectrometry analyses

The best emulsifier-producing strains were selected to carry out the quantification of diesel degradation. Gasoline experiments were discarded due to its high evaporation rate which resulted in the demise of the substrate after 1-week incubation at 30 °C. Therefore, 10 mL precultures of the selected strains were prepared (in the appropriate, LB or R2A, medium) and incubated overnight at 30 °C. The cultures were harvested by centrifugation and resuspended in the appropriate amount of liquid Bushnell Hass medium (BH, composition in g/L: 0.2 MgSO_4_, 0.02 CaCl_2_, 1.0 KH_2_PO_4_, 1.0 K_2_HPO_4_, 1.0 NH_4_NO_3_, 0.05 FeCl_3_; adjusted to pH 7) to obtain a cell concentration of OD_600_ = 1. Then, 3 mL of each culture were transferred to glass tests tubes and 25 µL of diesel were added to each tube and the assay was performed in triplicate. The corresponding controls were also prepared: sterile BH medium, sterile BH medium supplemented with diesel, and OD_600_ = 1 culture in BH medium without diesel. The cultures were incubated at 30 °C and 200 rpm agitation for 7 days. After the incubation period, the cultures with clear turbidity were selected for further study and the remaining diesel was analyzed by gas chromatography coupled to mass spectrometry (GC-MS). For liquid-liquid extraction of diesel, 2.5 mL of hexane were added to the assay tubes. Then, the tubes were agitated at a high speed in the vortex for 1 min and let rest for 15 min in a vertical-stable position, to allow the stable separation of both, the organic and aqueous phases. From the inoculated tubes as well as the negative control (BH medium supplemented with diesel) which was not subjected to bacterial degradation, 1 mL of the organic phase was recovered, filtered, and then analyzed by gas chromatography (Agilent Technologies® Model 7890B) using a 5MS UI column (Agilent, 5% phenyl and 95% polydimethylsiloxane; 30 m × 0.25 mm × 0.25 µm film thickness). The injection volume was 1 µL and the injection mode was Splitless. The temperature was set at 35 °C for 1 min; risen to 40 °C in 5 min (heating ramp of 10 °C/min); increased to 250 °C during 5 min (heating ramp of 40 °C/min); and finally risen to 300 °C during 10 min (heating ramp of 40 °C/min). The carrier gas was helium with a constant flow rate of 1 mL/min. The GC was coupled to a mass spectrometer (Agilent Technologies^®^ Model 5977 A) to identify the chromatographic peaks. The mass spectra were obtained by electronic impact ionization (EI) at 70 eV. Finally, the peak identification was carried out by the comparison with commercial libraries (Nis 11t, Nist_msms, mainlib, replib, and wiley7n). Software Mass Hunter Analysis (Agilent technologies^®^) was used for data acquisition and processing. Peaks show the diesel profile and the intensity of the peak is proportional to its abundance in the sample. All the samples were analyzed consecutively in the same conditions and in triplicate, hence, they are comparable and semiquantitative quantification can be extracted.

### Whole-genome sequencing of *Isoptericola* sp. 4D.3 and functional annotation

Genomic DNA extraction was carried out from fresh biomass of a pure culture of *Isoptericola* sp. 4D.3 by using the NZY Microbial gDNA Isolation kit (NZYTech, Cat. MB21702).

The Qubit x1 dsDNA HS Assay kit (Qubit 2.0 Fluorometer, Thermo Fisher, Waltham, USA) was used for DNA quantification. Genomic DNA was fragmented by sonication. After end polishing, full-length Illumina adapters were ligated. Then, PCR amplification with P5 and indexed P7 oligos was performed. Libraries were purified using the AMPure XP system (Beckman Coulter, CA, USA) and the Agilent 2100 Bioanalyzer (Agilent Technologies, CA, USA) was applied to check the size distribution. Finally, libraries were sequenced at Novogene UK (Cambridge Sequencing Center, UK) using the NovaSeq 6000 Illumina platform. Adapters were trimmed from the raw reads, and quality filtering was applied with BBDuk (BBTools v.38.84; Bushnell B., https://sourceforge.net/projects/bbtools/). Reads shorter than 75 bp and/or with a mean quality lower than Q20 (in the PHRED scale) were discarded. Bases with a quality below Q20 were also trimmed from both read ends. Sequencing adapters were removed with cutadapt (v. 3.4; https://cutadapt.readthedocs.io/en/stable/), while human sequences were discarded by aligning the reads with bowtie2 (v. 2.4.4; https://github.com/BenLangmead/bowtie2) to the human genome (GRCh38). The quality of the clean sequences was checked with FastQC (v0.11.5; http://www.bioinformatics.babraham.ac.uk/projects/fastqc). SPAdes (v. v3.14.1)^81^ was used for assembling the reads using the “--isolate” mode. Assembly statistics were calculated with QUAST (v. 5.0.2)^82^ and the completeness and contamination of the genomes were evaluated with checkm “lineage_wf” (v1.1.3)^83^. The genome was annotated using prokka (v. 1.14.6)^84^, using the default parameters.

Additionally, the predicted coding sequences were reannotated by the BlastKOALA tool (https://www.kegg.jp/blastkoala/) to study the degradation pathways involved in hydrocarbon degradation. The same open reading frames predicted by prokka were analyzed through CANT-HYD^28^ to find genes involved in hydrocarbon degradation (Supplementary Dataset 3). Finally, the taxonomic affiliation of strain 4D.3 was evaluated with TYGS^26^. Genera related to the strain were identified based on digital DNA-DNA hybridization (dDDH). All the genomes from strains belonging to those genera that were available at the NCBI Genome Database (https://www.ncbi.nlm.nih.gov/genome/) were downloaded. Finally, average nucleotide identity (ANI) values were calculated with FastANI^27^.

### Reporting Summary

Further information on research design is available in the Nature Research Reporting Summary linked to this article.

## Supplementary information

Supplementary information

Supplementary Dataset 1

Supplementary Dataset 2

Supplemenatry Dataset 3

Reporting Summary

## Data Availability

The datasets generated for this study can be found in online repositories. Raw reads are available at NCBI’s Sequence Read Archive (SRA) (Bioproject Accession PRJNA740157). 16S rRNA sequences are available at: https://www.ncbi.nlm.nih.gov/genbank/, MZ562353-MZ562399. *Isoptericola* sp. 4D.3 genome sequencing is available under the BioSample number SUB11086493.

## Code availability

All the code used in this study for data analysis has been uploaded to GitHub: https://github.com/adlape95/Car-Tank-Lid-Bacteriome
