# Supplementary material for: The car tank lid bacteriome: a reservoir of bacteria with potential in bioremediation of fuel
Source: NPJ Biofilms Microbiomes. 2022 Apr 28;8:32. doi: 10.1038/s41522-022-00299-8 (PMC9050737; doi:10.1038/s41522-022-00299-8)
Supplement: Supplementary file 1 — Supplementary information [file 41522_2022_299_MOESM1_ESM.pdf]

Supplementary information

“The car tank lid bacteriome”

Àngela Vidal-Verdú[†], Daniela Gómez-Martínez[†], Adriel Latorre-Pérez, Juli Peretó, Manuel Porcar[*]

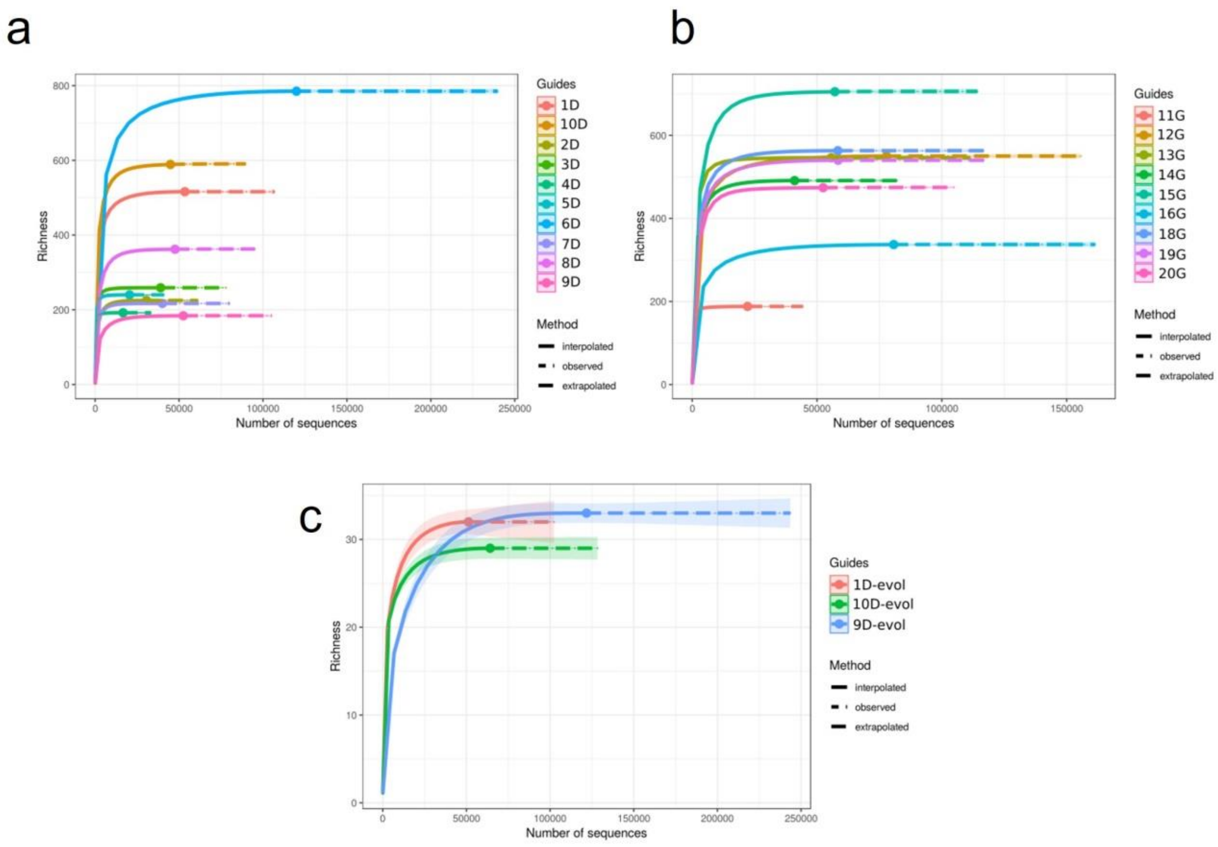

Supplementary figure 1. Rarefaction curves of **a)** Diesel cars **b)** Gasoline vehicles and **c)** Enriched samples with diesel as sole carbon source.

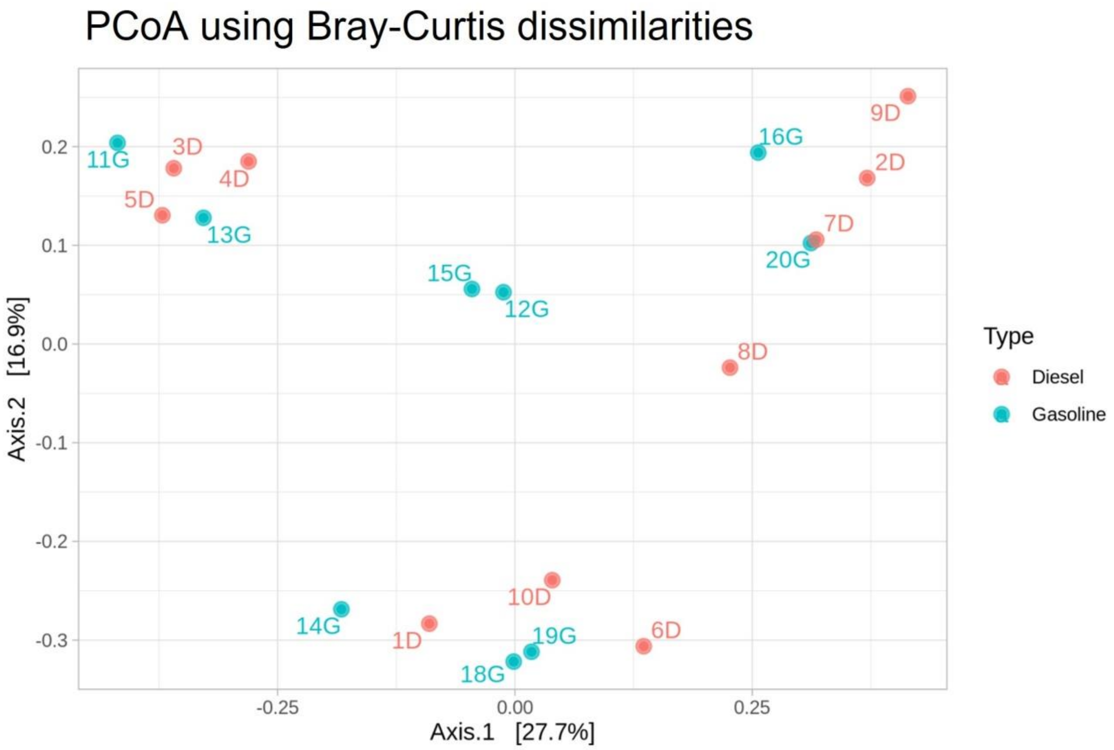

Supplementary figure 2. Heterogeneous distribution of the bacterial communities of samples taken from car tank lids. Variations between samples taken from cars fueled with diesel (red) and cars fueled with gasoline (blue) can be observed.

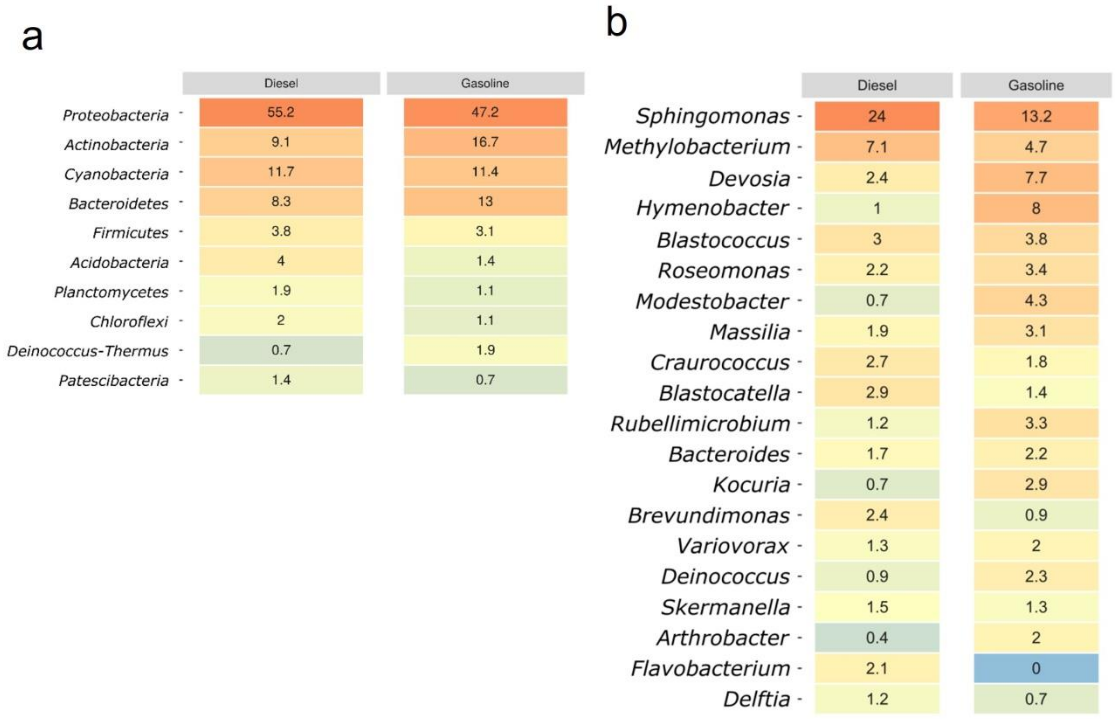

Supplementary figure 3. Ten bacterial phyla **a)** and twenty bacterial genera **b)** with the highest mean abundance.

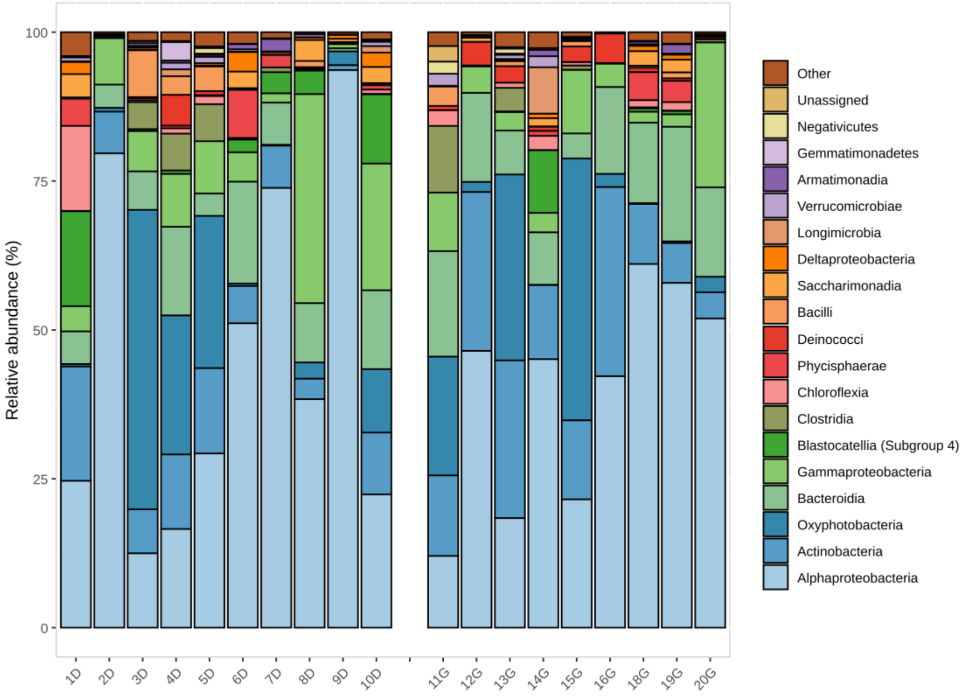

Supplementary figure 4. Barplot showing bacterial class diversity of diesel samples (1D-10D) and gasoline samples (11G-20G).

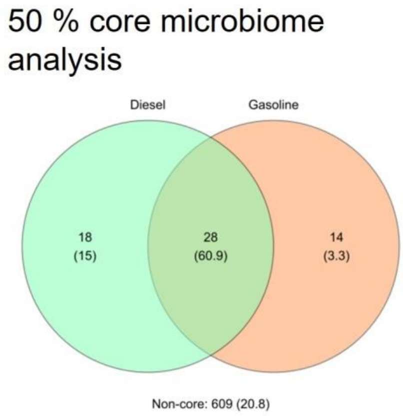

Supplementary figure 5. Core microbiome at 50% sample representation.

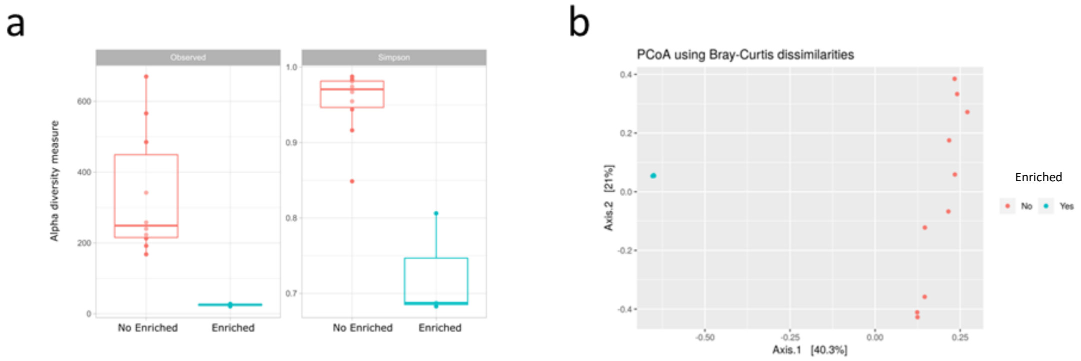

Supplementary figure 6. a) Richness (“Observed”) and Simpson diversity of enriched and not enriched samples **b)** PCoA comparing enriched and not enriched samples. Center line = median; bounds of box = Q1 and Q3; lower whisker = smallest observation greater than or equal to lower hinge - 1.5 * IQR ; higher whisker = largest observation less than or equal to upper hinge + 1.5 * IQR; Q = quartile ; IQR = interquartile ra

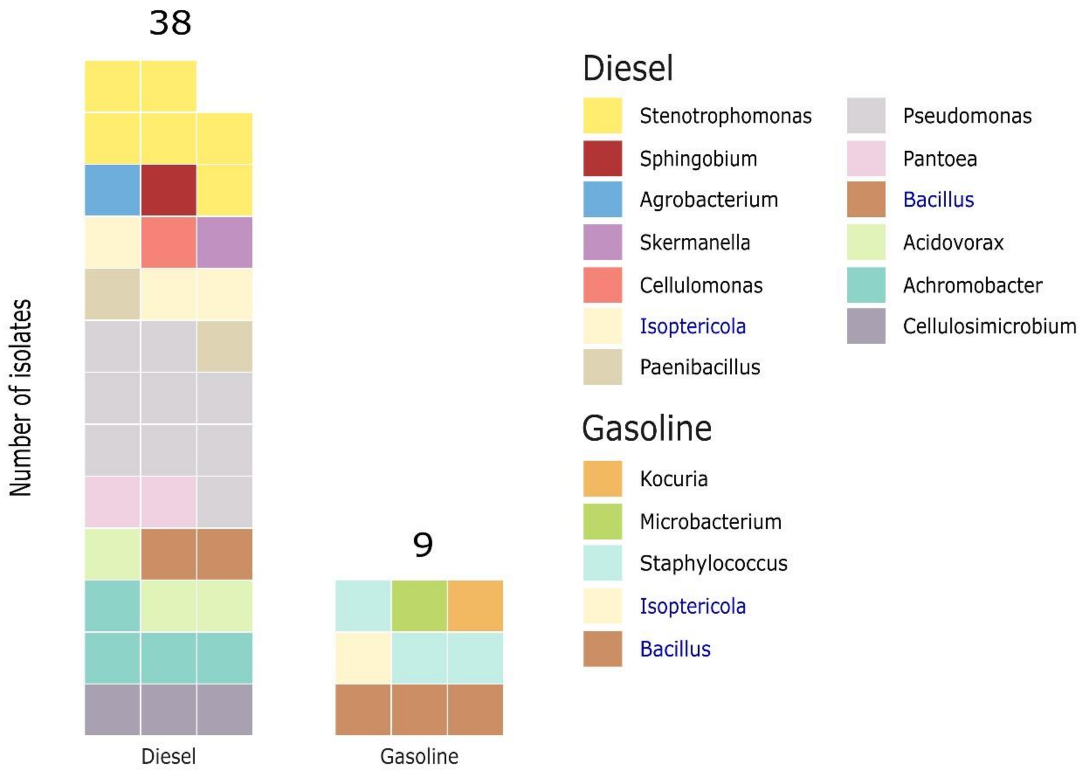

Supplementary figure 7. **Genera collection of culturable strains from diesel- and gasoline-enriched cultures.** Each square represents one isolate and the difference in number of isolates is shown, 39 isolates from diesel cultures and 9 isolates from gasoline cultures. Common genera in both, gasoline and diesel containing cultures, are colored in blue in the caption.

a.

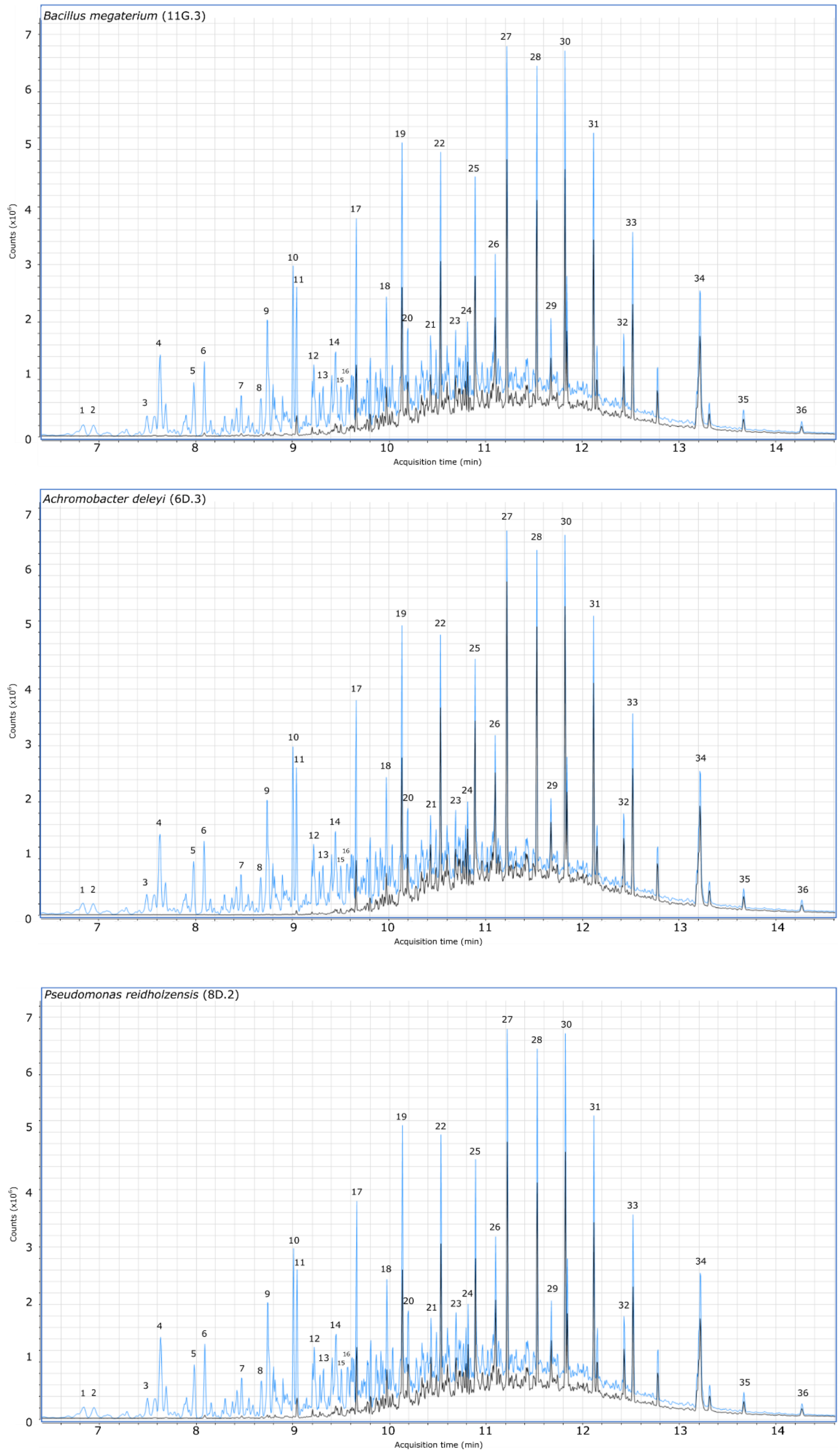
Bacillus megaterium (11G.3)
Counts (x10^6)
Acquisition time (min)
Achromobacter deleyi (6D.3)
Counts (x10^6)
Acquisition time (min)
Pseudomonas reidholzensis (8D.2)
Counts (x10^6)
Acquisition time (min)

b. *Isoptericola* sp. (4D.3)

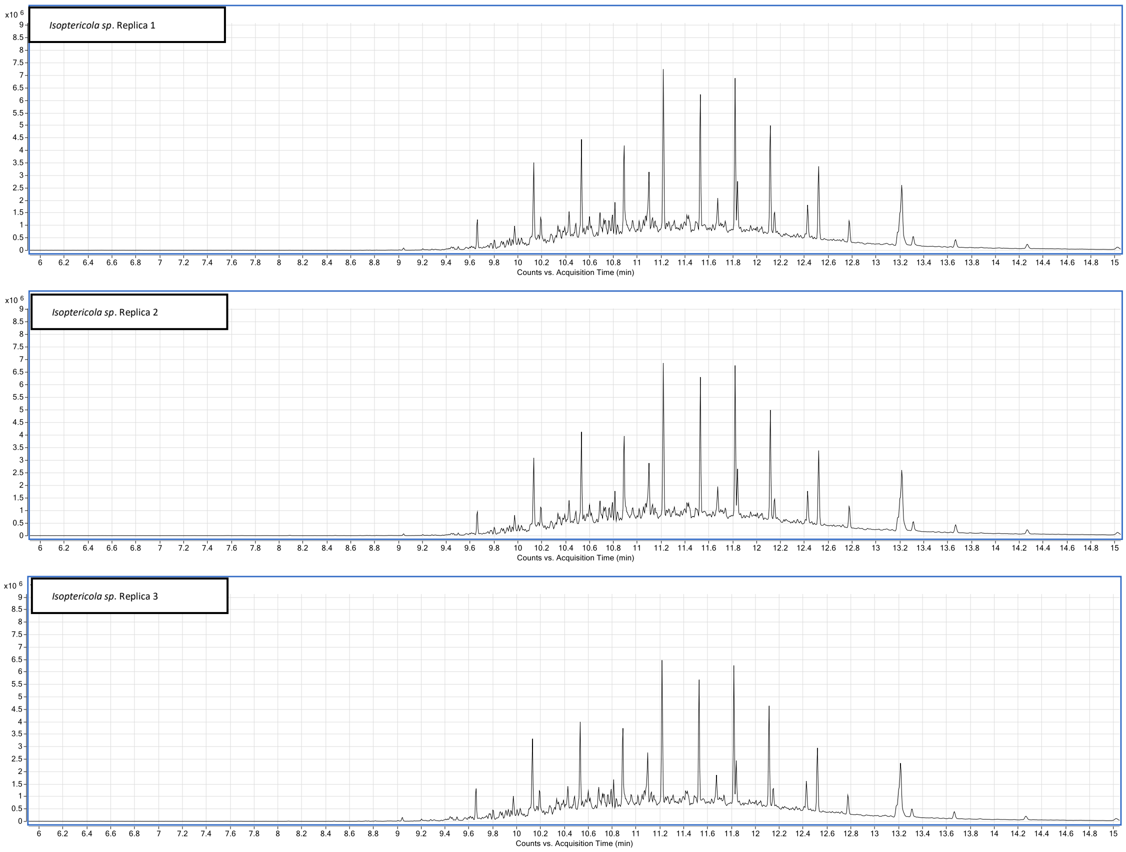

c. *Achromobacter deleyi* (6D.3)

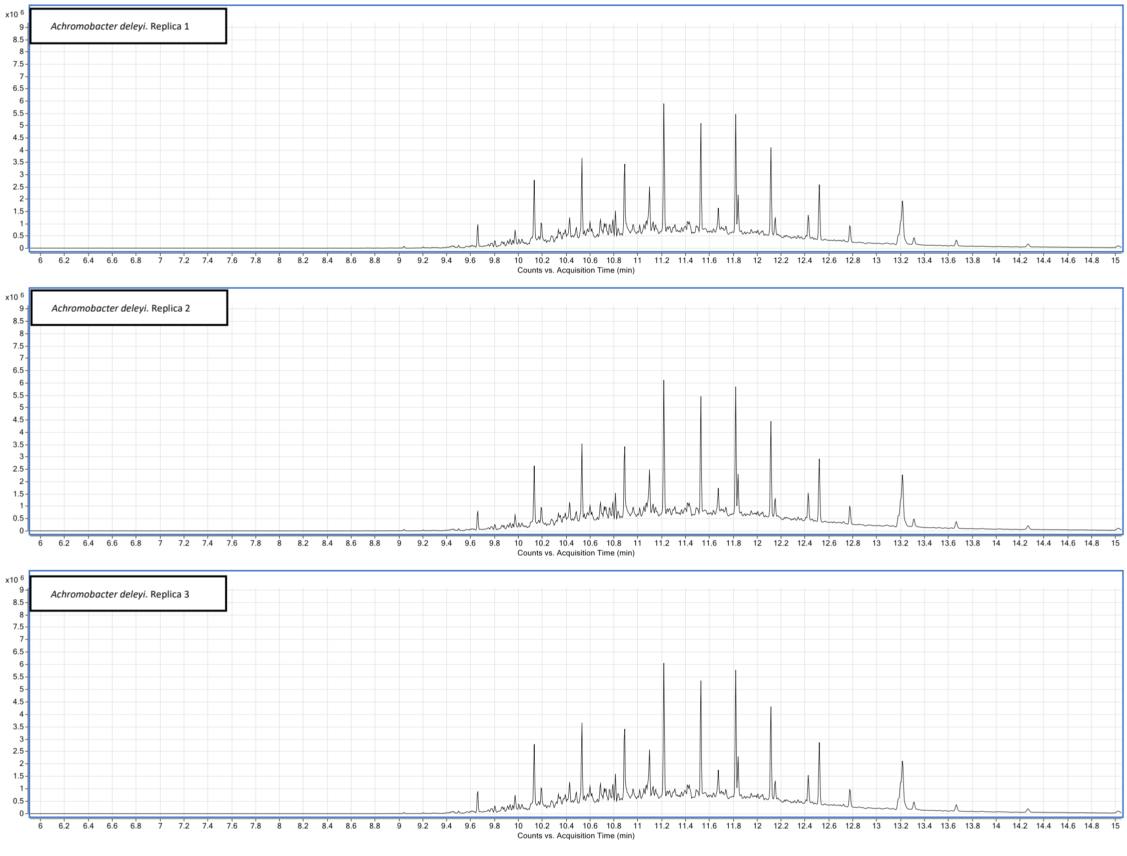

d. *Pseudomonas reidholzensis* (8D.2)

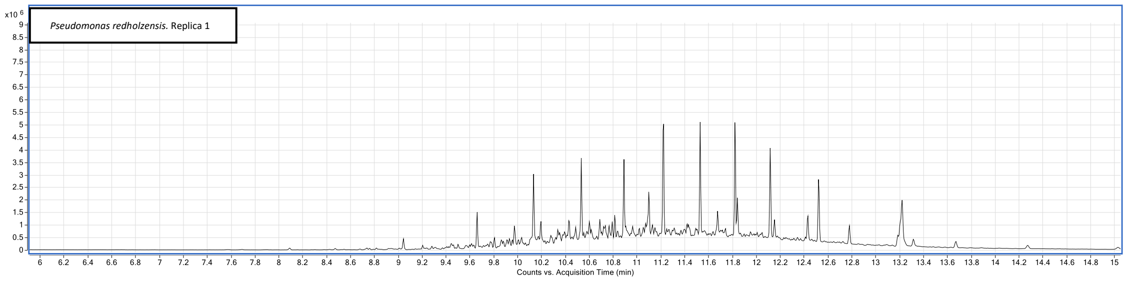

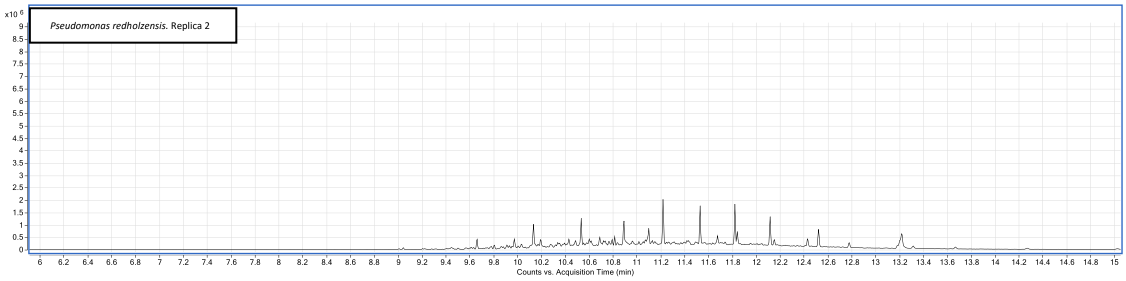

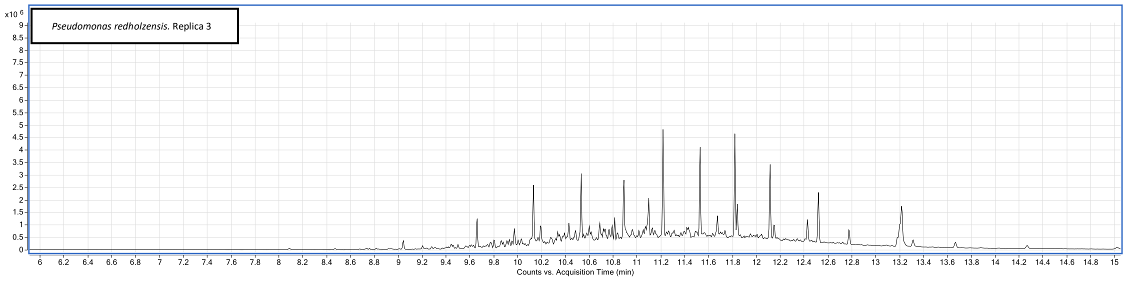

e. *Pseudomonas lutea* (9D.7)

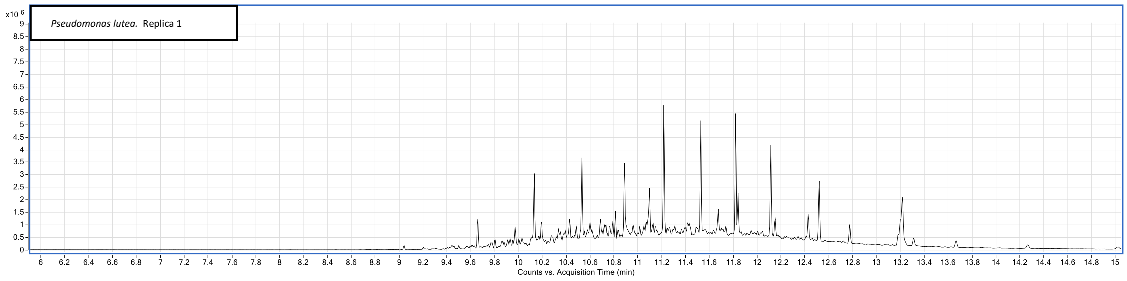

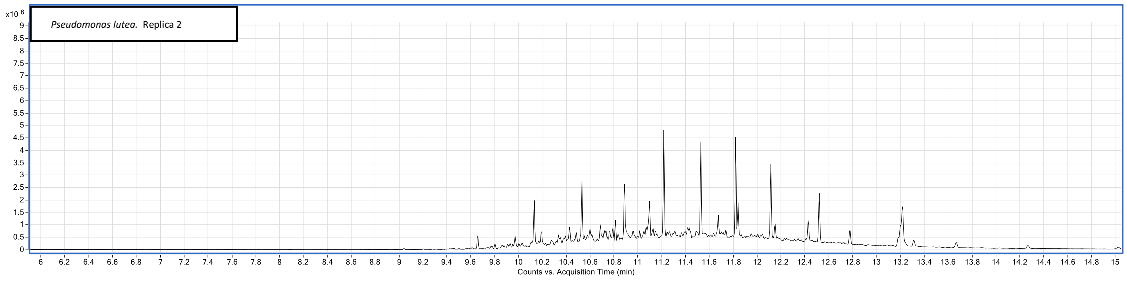

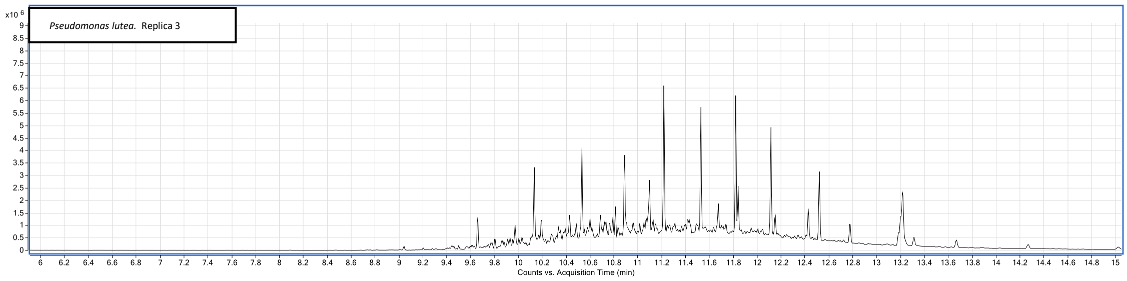

f. *Bacillus megaterium (*11G.3)

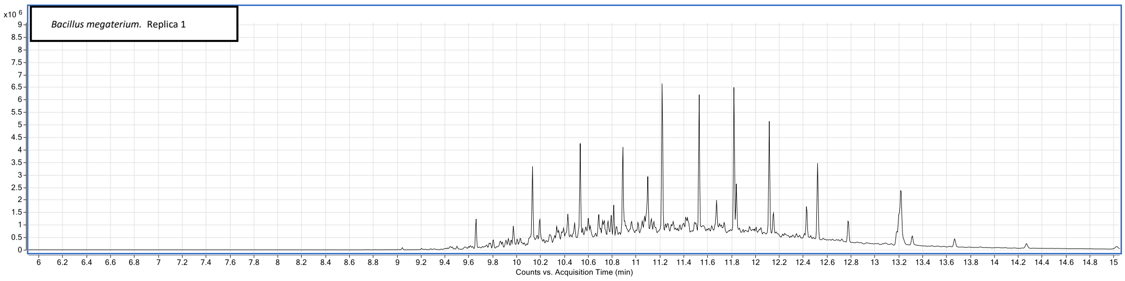

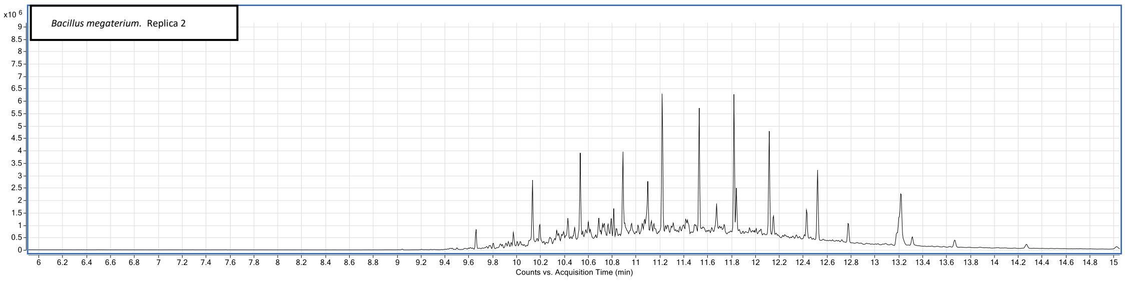

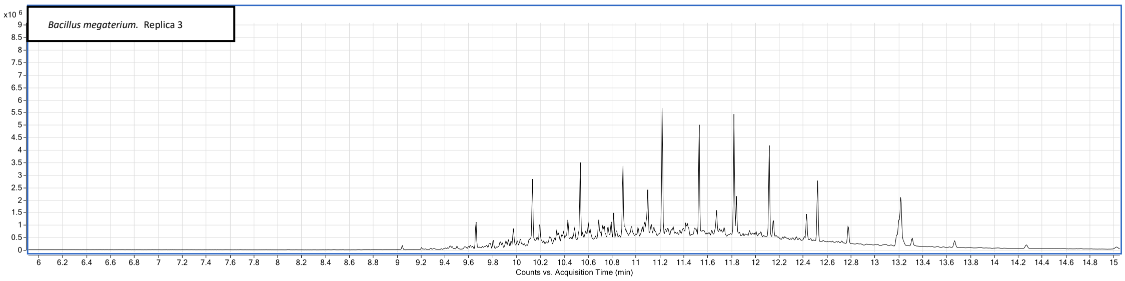

g. Control samples

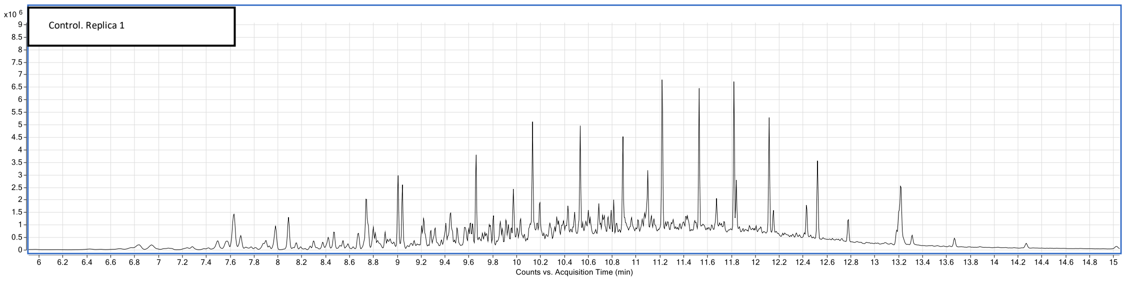

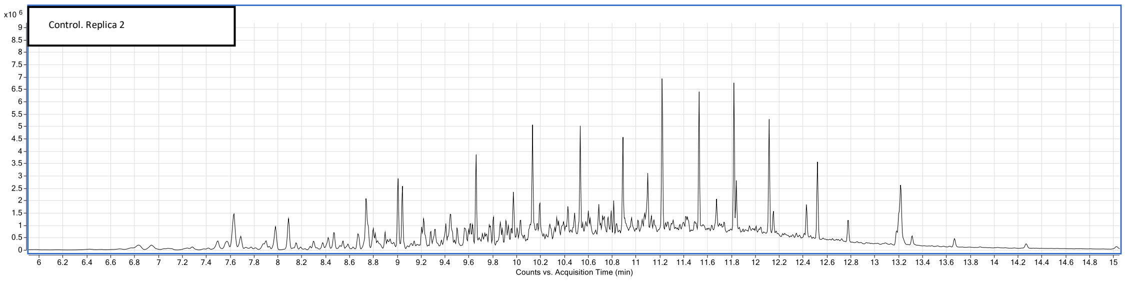

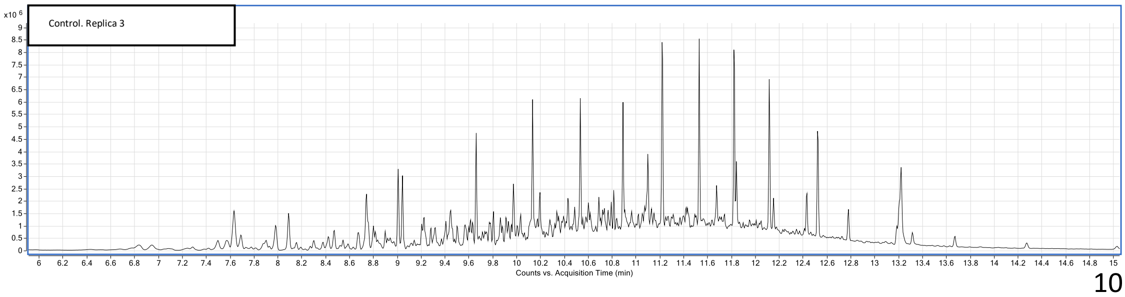

Supplementary figure 8. Gas chromatograms of diesel extracted from cultures inoculated with diesel degrading bacteria. **a)** Control sample (blue) and inoculated sample (black) are overlaped for *Achromobacter deleyi*, *Bacillus megaterium* and *Pseudomonas reidholzensis*. **b-g)** Individual chromatograms for each sample and each replica.

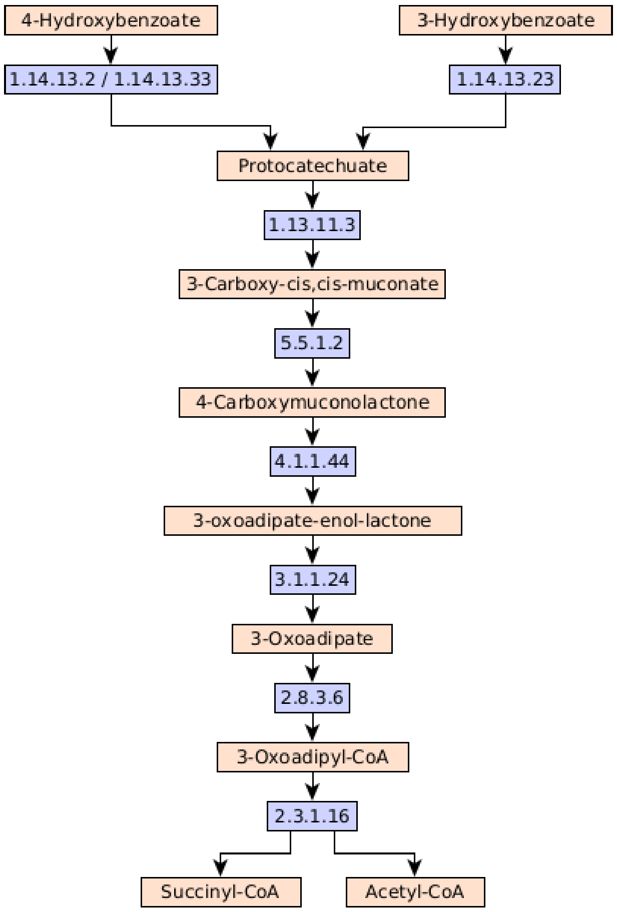

Supplementary figure 9. Aromatic degradation pathway present in the strain *Isoptericola* sp. 4D.3. The complete catabolic pathway from 4-hydroxybenzoate and 3-hydroxybenzoate to central metabolites has been found in the functional annotation of the genome. The corresponding EC number for enzymatic activities is shown at each step of the pathway. The nucleotide sequence from *Isoptericola* sp. 4D.3 genome of each enzyme can be checked at Suplementary Dataset 2.

Supplementary table 1. Strain collection identified by amplification of the gene coding for 16S rRNA gene and subsequent alignment with sequences of each strain integrated into databases. Vehicle of origin (G: gasoline; D: Diesel), as well as the name of the complete taxon and the strain is specified. Also, the percentage of similarity of the amplicon with the sequences in the database and the Genbank accession number for deposited sequences.

**Sample**	**Closest Type Strain**	**Accession number**	**ID %**	**GenBank accession number**
1D.1	*Cellulosimicrobium cellulans*	CAOI01000359	99.54	MZ562353
1D.2	*Pantoea agglomerans*	AJ233423	100	MZ562354
1D.3	*Cellulosimicrobium cellulans*	CAOI01000359	99.85	MZ562355
2D.1	*Sphingobium yanoikuyae*	JH992904	98.99	MZ562356
2D.2	*Pseudomonas juntendi*	MK680061	98.82	MZ562357
2D.3	*Cellulomonas pakistanensis*	BBHV01000063	99.38	MZ562358
3D.1	*Acidovorax avenae*	CP002521	99.26	MZ562359
3D.2	*Acidovorax avenae*	CP002521	99.12	MZ562360
3D.3	*Acidovorax avenae*	CP002521	99.6	MZ562361
4D.2	*Isoptericola nanjingensis*	HQ222356	99.47	MZ562362
4D.3	*Isoptericola nanjingensis*	HQ222356	99.66	MZ562363
4D.4	*Isoptericola nanjingensis*	HQ222356	99.6	MZ562364
5D.1	*Skermanella aerolata*	DQ672568	99.25	MZ562365
6D.1	*Stenotrophomonas rhizophila*	CP007597	100	MZ562366

**Sample**	**Closest Type Strain**	**Accession number**	**ID %**	**GenBank accession number**
6D.2	*Stenotrophomonas rhizophila*	CP007597	100	MZ562367
6D.3	*Achromobacter deleyi*	HG324053	100	MZ562368
6D.4	*Stenotrophomonas rhizophila*	CP007597	100	MZ562369
7D.1	*Stenotrophomonas bentonitica*	LT622838	99.14	MZ562370
7D.2	*Paenibacillus lautus*	BIMF01000051	99.27	MZ562371
7D.3	*Paenibacillus lautus*	BIMF01000051	99.44	MZ562372
8D.1	*Stenotrophomonas rhizophila*	CP007597	99.69	MZ562373
8D.2	*Pseudomonas reidholzensis*	LT009707	99.23	MZ562374
8D.3	*Pseudomonas koreensis*	AF468452	99.85	MZ562375
8D.4	*Pseudomonas koreensis*	AF468452	99.86	MZ562376
8D.5	*Pseudomonas koreensis*	AF468452	99.86	MZ562377
9D.1	*Pseudomonas lutea*	JRMB01000004	100	MZ562378
9D.2	*Pseudomonas lutea*	JRMB01000004	100	MZ562379
9D.3	*Bacillus cereus*	AE016877	100	MZ562380
9D.4	*Stenotrophomonas rhizophila*	CP007597	99.62	MZ562381
9D.5	*Bacillus cereus*	AE016877	100	MZ562382
9D.6	*Pseudomonas lutea*	JRMB01000004	100	MZ562383
9D.7	*Pseudomonas lutea*	JRMB01000004	100	MZ562384
10.D1	*Agrobacterium arsenijevicii*	JWIT01000061	99.33	MZ562385
10.D2	*Pantoea eucalypti*	EF688009	99.63	MZ562386
10.D3	*Achromobacter mucicolens*	HE613446	99.54	MZ562387

**Sample**	**Closest Type Strain**	**Accession number**	**ID %**	**GenBank accession number**
10.D4	*Achromobacter mucicolens*	HE613446	99.49	MZ562388
10.D5	*Achromobacter mucicolens*	HE613446	99.52	MZ562389
10.D6	*Cellulosimicrobium cellulans*	CAOI01000359	99.51	MZ562390
11G.1	*Bacillus megaterium*	JJMH01000057	100	MZ562391
11G.2	*Bacillus megaterium*	JJMH01000057	100	MZ562392
11G.3	*Bacillus megaterium*	JJMH01000057	100	MZ562393
15G.1	*Staphylococcus edaphicus*	KY315825	100	MZ562394
15G.2	*Microbacterium testaceum*	BJML01000022	99.64	MZ562395
19G.1	*Staphylococcus pasteuri*	AF041361	99.85	MZ562396
19G.2	*Staphylococcus pasteuri*	AF041361	99.84	MZ562397
19G.3	*Isoptericola nanjingensis*	HQ222356	99.81	MZ562398
19G.4	*Kocuria arsenatis*	KM874399	99.82	MZ562399

Supplementary Table 2. Compound identification of the peaks in the diesel GC by comparison with commercial libraries (Nis 11t, Nist_msms, mainlib, replib and wiley7n). The number of the peak correspond to the peak numbering in Figure 6 and Supplementary Figure 8-a.

Peak	RT	NAME	FORMULA	STRUCTURE
1	6.84	(E)-4-Oxohex-2-enal	C6H8O2	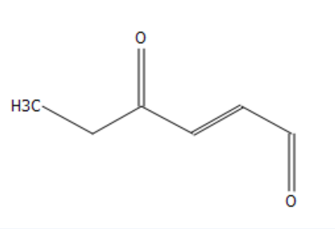
2	6.94	1,1,4-Trimethylcyclohexane	C9H18	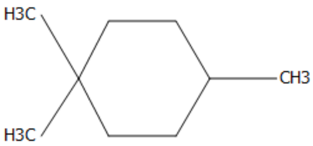
3	7.5	Ethylbenzene	C8H10	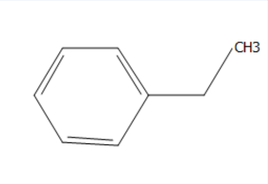
4	7.63	Benzene, 1,3-dimethyl	C8H10	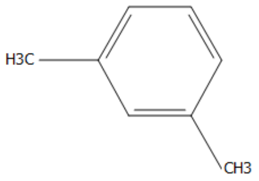
5	7.98	p-Xylene	C8H10	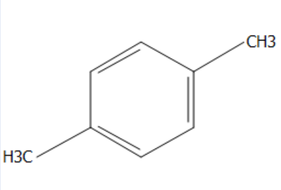
6	8.09	4,4-Dimethyl octane	C10H22	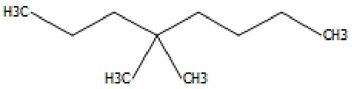
7	8.47	Dodecane, 1-fluoro	C12H25F	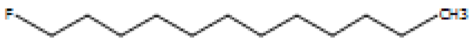

Peak	RT	NAME	FORMULA	STRUCTURE
8	8.68	Carbonic acid, decyl ethylester	C13H26O3	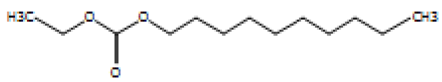
9	8.74	Benzene, 1,2,3-trimethyl	C9H12	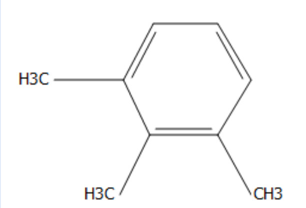
10	9.01	Benzene, 1,2,4-trimethyl	C9H12	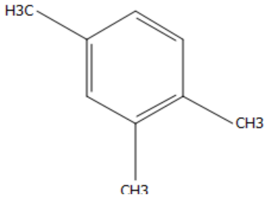
11	9.04	Octane, 3,5-dimethyl	C10H22	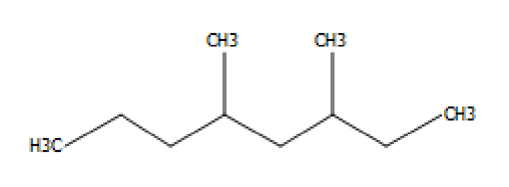
12	9.22	Mesitylene	C9H12	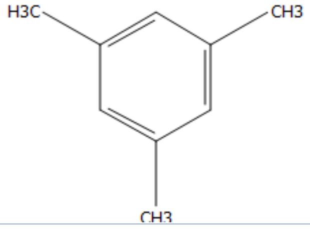
13	9.32	Indane	C9H10	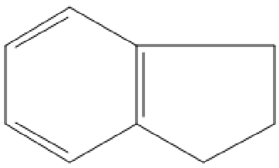
14	9.45	Carveol	C10H16O	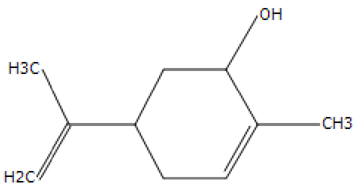
15	9.5	Propyl undecyl carbonate	C15H30O3	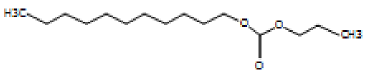

Peak	RT	NAME	FORMULA	STRUCTURE
16	9.57	p-Cymene	C10H14	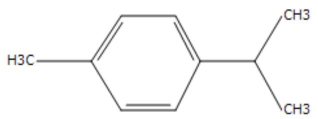
17	9.66	Nonane, 4,5-dimethyl	C11H24	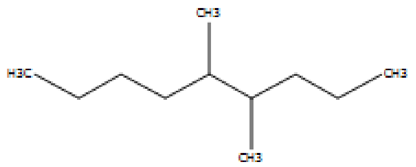
18	9.97	Carveol	C10H16O	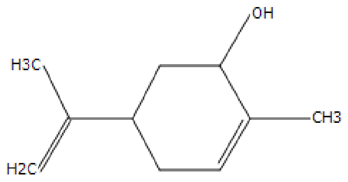
19	10.13	1-Decanol, 2-hexyl	C16H34O	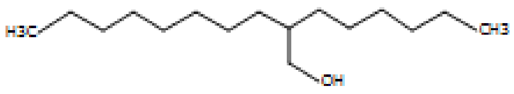
20	10.2	cis-1-Chloro-9-octadecene	C18H35Cl	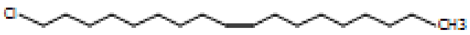
21	10.43	cis-1-Chloro-9-octadecene	C18H35Cl	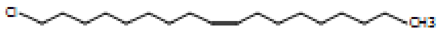
22	10.53	1-Decanol, 2-hexylp	C16H34O	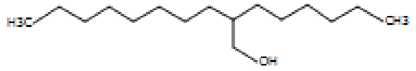
23	10.69	Naphthalene, 1,2,3,4-tetrahydro-1,5-dimethyl	C12H16	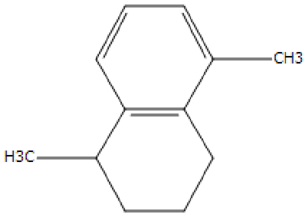
24	10.81	Octadecane, 1-chloro-	C18H37Cl	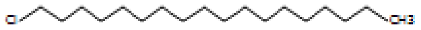
25	10.89	tert-Hexadecanethiol	C16H34S	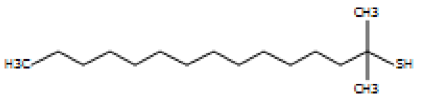

Peak	RT	NAME	FORMULA	STRUCTURE
26	11.1	Falcarinol	C17H24O	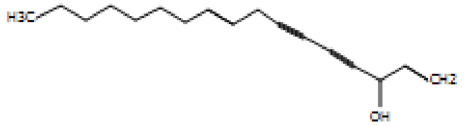
27	11.22	Pentadecane	C15H32	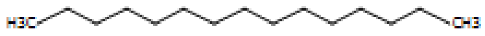
28	11.53	Hexadecane	C16H34	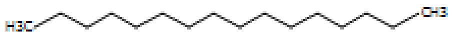
29	11.67	2-Methyl-cis-7,8-epoxynonadecane	C20H40O	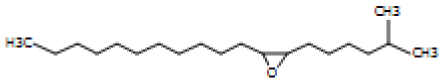
30	11.82	Heptadecane	C17H36	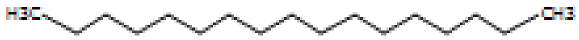
31	12.12	Octadecane	C18H38	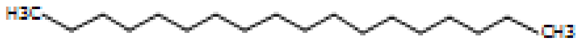
32	12.43	Nonadecane	C19H40	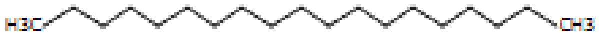
33	12.52	Hexadecanoic acid, methyl ester	C17H34O2	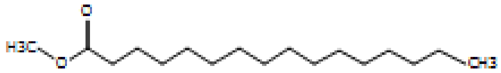
34	13.22	9-Octadecenoic acid (Z)-,methyl ester	C19H36O2	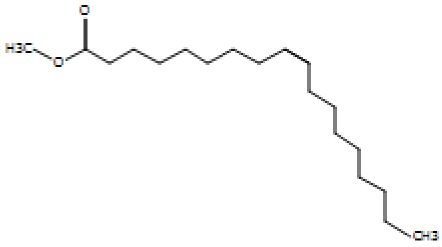

Peak	RT	NAME	FORMULA	STRUCTURE
35	13.67	2-Methyl-cis-7,8-epoxynonadecane	C20H40O	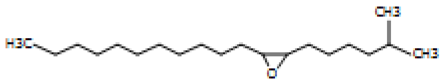
36	14.27	Hexadecane, 2,6,10,14-tetramethyl	C20H42	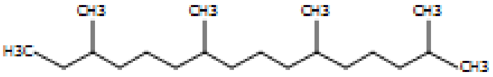
