# Supplementary material for: The car tank lid bacteriome: a reservoir of bacteria with potential in bioremediation of fuel
Source: NPJ Biofilms Microbiomes. 2022 Apr 28;8:32. doi: 10.1038/s41522-022-00299-8 (PMC9050737; doi:10.1038/s41522-022-00299-8)
Supplement: Supplementary file 5 — Reporting Summary [file 41522_2022_299_MOESM5_ESM.pdf]

nature portfolio

Corresponding author(s): Manuel Porcar

Last updated by author(s): Mar 29, 2022

Reporting Summary

Nature Portfolio wishes to improve the reproducibility of the work that we publish. This form provides structure for consistency and transparency in reporting. For further information on Nature Portfolio policies, see our Editorial Policies and the Editorial Policy Checklist.

Statistics

For all statistical analyses, confirm that the following items are present in the figure legend, table legend, main text, or Methods section.

n/a	Confirmed	
☐	☒	The exact sample size (*n*) for each experimental group/condition, given as a discrete number and unit of measurement
☐	☒	A statement on whether measurements were taken from distinct samples or whether the same sample was measured repeatedly
☐	☒	The statistical test(s) used AND whether they are one- or two-sided<br>*Only common tests should be described solely by name; describe more complex techniques in the Methods section.*
☒	☐	A description of all covariates tested
☐	☒	A description of any assumptions or corrections, such as tests of normality and adjustment for multiple comparisons
☒	☐	A full description of the statistical parameters including central tendency (e.g. means) or other basic estimates (e.g. regression coefficient) AND variation (e.g. standard deviation) or associated estimates of uncertainty (e.g. confidence intervals)
☒	☐	For null hypothesis testing, the test statistic (e.g. *F*, *t*, *r*) with confidence intervals, effect sizes, degrees of freedom and *P* value noted<br>*Give P values as exact values whenever suitable.*
☒	☐	For Bayesian analysis, information on the choice of priors and Markov chain Monte Carlo settings
☒	☐	For hierarchical and complex designs, identification of the appropriate level for tests and full reporting of outcomes
☒	☐	Estimates of effect sizes (e.g. Cohen's *d*, Pearson's *r*), indicating how they were calculated

Our web collection on statistics for biologists contains articles on many of the points above.

Software and code

Policy information about availability of computer code

Data collection	*Taxonomic profile of the car tank lids compared to other hydrocarbon-polluted environments:<br><br>Datasets were obtained based on a literature search in Scopus (www.scopus.com), by using the search line: ALL ("16S" AND "Illumina") AND (TITLE-ABS-KEY (hydrocarbon OR diesel OR gasoline OR oil AND contaminated OR polluted)), as well as a database search on the NCBI SRA by search with the following syntax: Hydrocarbon [All Fields] AND Polluted [All Fields] AND X metagenome [Organism], where X was "sediment" and "soil". To confirm the completeness of this search, an additional Google Scholar search was carried out using the search terms "hydrocarbon", "polluted" and "Illumina." The first 500 hits were reviewed for any studies that were not captured in the main searches. The aim of this search was to capture available datasets that describe hydrocarbon polluted soils and other environmental surfaces. Only those studies that used the V3-V4 region of the 16S rRNA gene for microbiome sequencing were included in the selection (Supplementary Dataset 3). Both polluted and non-polluted soil samples were considered in the analysis.
Data analysis	*16S rRNA gene sequencing and analysis of the originial samples in the study:<br><br>Raw reads were processed with Qiime2 (v.2019.4) 68. Briefly, the pipeline consisted of (1) the quality assessment via the demux plugin, (2) the error correction and the sequence variant clustering through DADA2, and (3) the assignment of the reads against SILVA database (v. 132)) using the classify-Sklearn module from the feature-classifier plugin. Principal Coordinates Analyses (PCoA) were carried out with phyloseq R package (v. 1.22.3) 69 using Bray-Curtis dissimilarities, and PERMANOVA tests were calculated with vegan (v. 2.5-3). Rarefaction curves were also constructed in R via iNEXT package (v. 2.0.17) 70. The core microbiome analyses were performed with the amp_venn function from the ampvis2 package (v. 2.6.5) (https://doi.org/10.1101/299537). DESeq2 (v. 1.26.0) 73 was used for differential abundance analyses.<br><br>*EzBioCloud 16S Database was used for 16S rRNA gene identification of strains.<br><br>*Taxonomic profile of the car tank lids compared to other hydrocarbon-polluted environments:

Bioinformatic analyses were carried out with QIIME2 71. All the samples included in a particular study or experiment were processed independently using the DADA2 plugin, as recommended by the authors of QIIME2 (https://docs.qiime2.org/2021.11/tutorials/fmt/). Taxonomy was assigned using the SILVA database (v. 138) 72. For alpha diversity, a rarefaction to 10,000 reads per sample was performed. Samples below this threshold were removed from the analysis. Beta diversity analysis was performed as described in the previous section. Finally, control samples were removed from the analysis and a core microbiome was calculated considering all the samples exposed to hydrocarbon pollution (Supplementary Table Dataset 3

For manuscripts utilizing custom algorithms or software that are central to the research but not yet described in published literature, software must be made available to editors and reviewers. We strongly encourage code deposition in a community repository (e.g. GitHub). See the Nature Portfolio guidelines for submitting code & software for further information.

Data

Policy information about availability of data

All manuscripts must include a data availability statement. This statement should provide the following information, where applicable:

- Accession codes, unique identifiers, or web links for publicly available datasets
- A description of any restrictions on data availability
- For clinical datasets or third party data, please ensure that the statement adheres to our policy

The datasets generated for this study can be found in online repositories. Raw reads are available at NCBI's Sequence Read Archive (SRA) (Bioproject Accession PRJNA740157). 16S rRNA sequences are available at: https://www.ncbi.nlm.nih.gov/genbank/, MZ562353-MZ562399. Isoptericola sp. 4D.3 genome sequencing is available under the BioSample number SUB11086493.

Field-specific reporting

Please select the one below that is the best fit for your research. If you are not sure, read the appropriate sections before making your selection.

☐ Life sciences ☐ Behavioural & social sciences ☒ Ecological, evolutionary & environmental sciences

For a reference copy of the document with all sections, see nature.com/documents/nr-reporting-summary-flat.pdf

Ecological, evolutionary & environmental sciences study design

All studies must disclose on these points even when the disclosure is negative.

Study description	In this study the characterization of the bacterial communities inhabiting the car tank lids of gasoline and diesel has been carried out by a dual perspective. On the one hand, 16S rRNA gene sequencing (culture-independent) was performed to compare the bacterial community from 10 diesel fueled cars and 10 gasoline fueled cars. On the other hand, a culture-dependent approach was carried out to enrich the samples in fuel-degrading strains as well as the quantification of the degradation from some isolates by GC-MS.
Research sample	The research samples in this study consisted of the black dust totally or partially soaked with fuel residues from the car tank lids of 10 diesel deposits and 10 gasoline deposits.
Sampling strategy	The aim of this study was to compare whether the bacterial communities thriving in diesel or gasoline car tank lids were different. Therefore, the number of total samples was 20: 10 replicates from diesel-fueled cars and 10 replicates from gasoline-fueled cars.
Data collection	Àngela Vidal-Verdú and Daniela Gómez-Martínez performed the sample collection. Samples were taken by a sterile handle and deposited in a 1.5 mL sterile tubes and transported to the laboratory for further processing steps. Each car age and brand was written down.
Timing and spatial scale	All the samples were taken between the second and third of July 2018 with no spatial scale. They were taken from cars at the parking areas of The Institute for Integrative Systems Biology and the Polytechnic University of Valencia, both in Valencia, Spain.
Data exclusions	All the samples were procesed the same way in this study in order to extract the bacterial DNA. However, sample 17G did not yield enough DNA concentration to carry out the 16S rRNA gene sequencing, thus was discarded.
Reproducibility	For DNA extraction and 16S rRNA gene sequencing just one replicate from a gasoline-fueled car failed and the rest performed quite similar in terms of DNA concentration and sequencing quality. For the quantification of diesel degradation by GC-MS the experiment was carried out in triplicate and results are very consistent among them.
Randomization	Samples were taken from two different parking areas. Therefore, 5 gasoline-fueled cars and 5 diesel-fueled cars were sampled in each location to distribute equally the number of samples from each fuel type and parking area.
Blinding	The cars sampled were selected randomly.

Did the study involve field work? ☒ Yes ☐ No

Field work, collection and transport

Field conditions	Sampling was carried out in July 2018. It was sunny and the temperature was 26-29 degree Celcius.
Location	Two different parking areas were chosen to carry out the sampling. On the one hand, the parking area of the Institute for Integrative Systems Biology (39° 30' 36'' N, 0° 25' 12.0'' W) and, on the other hand, from the parking area of the Polytechnic University of Valencia (39° 28' 55.2'' N, 0° 20' 20.4'' W).
Access & import/export	The owners of the cars agreed to participate in the study.
Disturbance	There were no disturbances during the study.

Reporting for specific materials, systems and methods

We require information from authors about some types of materials, experimental systems and methods used in many studies. Here, indicate whether each material, system or method listed is relevant to your study. If you are not sure if a list item applies to your research, read the appropriate section before selecting a response.

Materials & experimental systems

n/a	Involved in the study	
☒	☐	Antibodies
☒	☐	Eukaryotic cell lines
☒	☐	Palaeontology and archaeology
☒	☐	Animals and other organisms
☒	☐	Human research participants
☒	☐	Clinical data
☒	☐	Dual use research of concern

Methods

n/a	Involved in the study	
☒	☐	ChIP-seq
☒	☐	Flow cytometry
☒	☐	MRI-based neuroimaging
